# Enabling personalised disease diagnosis by combining a patient’s time-specific gene expression profile with a biomedical knowledge base

**DOI:** 10.1186/s12859-024-05674-0

**Published:** 2024-02-07

**Authors:** Ghanshyam Verma, Dietrich Rebholz-Schuhmann, Michael G. Madden

**Affiliations:** 1 Insight Centre for Data Analytics, School of Computer Science, University of Galway, Galway, Ireland; 2 School of Computer Science, University of Galway, Galway, Ireland; 3 ZB MED - Information Centre for Life Sciences, University of Cologne, Cologne, Germany

**Keywords:** Machine learning, Knowledge base, Gene expression, Respiratory viral infection, Personalized disease diagnosis

## Abstract

**Background:**

Recent developments in the domain of biomedical knowledge bases (KBs) open up new ways to exploit biomedical knowledge that is available in the form of KBs. Significant work has been done in the direction of biomedical KB creation and KB completion, specifically, those having gene-disease associations and other related entities. However, the use of such biomedical KBs in combination with patients’ temporal clinical data still largely remains unexplored, but has the potential to immensely benefit medical diagnostic decision support systems.

**Results:**

We propose two new algorithms, LOADDx and SCADDx, to combine a patient’s gene expression data with gene-disease association and other related information available in the form of a KB, to assist personalized disease diagnosis. We have tested both of the algorithms on two KBs and on four real-world gene expression datasets of respiratory viral infection caused by Influenza-like viruses of 19 subtypes. We also compare the performance of proposed algorithms with that of five existing state-of-the-art machine learning algorithms (*k*-NN, Random Forest, XGBoost, Linear SVM, and SVM with RBF Kernel) using two validation approaches: LOOCV and a single internal validation set. Both SCADDx and LOADDx outperform the existing algorithms when evaluated with both validation approaches. SCADDx is able to detect infections with up to 100% accuracy in the cases of Datasets 2 and 3. Overall, SCADDx and LOADDx are able to detect an infection within 72 h of infection with 91.38% and 92.66% average accuracy respectively considering all four datasets, whereas XGBoost, which performed best among the existing machine learning algorithms, can detect the infection with only 86.43% accuracy on an average.

**Conclusions:**

We demonstrate how our novel idea of using the most and least differentially expressed genes in combination with a KB can enable identification of the diseases that a patient is most likely to have at a particular time, from a KB with thousands of diseases. Moreover, the proposed algorithms can provide a short ranked list of the most likely diseases for each patient along with their most affected genes, and other entities linked with them in the KB, which can support health care professionals in their decision-making.

**Supplementary Information:**

The online version contains supplementary material available at 10.1186/s12859-024-05674-0.

## Background

Due to advances in the field of genomics in the past two decades, the focus of medical science has been shifting from disease-centric to person-centric diagnostic and therapeutic methods [1, 2]. The development of microarray techniques and new advances in RNA sequencing have improved our ability to explore the underlying molecular mechanisms associated with complex diseases [3]. Gene expression profiles are being used to identify disease-specific genome-wide changes in genes, which can help in the identification of differentially expressed genes (DEGs): these are genes whose expression levels significantly differ between the healthy state and the diseased state [4, 5]. The motivation behind the identification of DEGs is to understand the molecular processes involved in the progression of a disease. These DEGs can be used as important biomarkers for patient classification [3, 6], disease diagnosis [7], and drug target identification [8].

A knowledge base is an extensive collection of structured or unstructured data that represent facts about the world [9, 10]. It is a dataset with some formal semantics that may contain different kinds of knowledge, for example, facts, rules, axioms, statements, definitions, and primitives [11, 12]. Although some researchers have used the terms ‘knowledge base’ and ‘knowledge graph’ interchangeably, e.g. [13–16], the use of ‘graph’ generally implies that it has some specific features. The fundamental factor that sets knowledge graphs apart from knowledge bases lies in their emphasis on the interconnectedness of entities, reasoning capabilities, and graph structure [12, 14, 17]. While all knowledge graphs can be considered knowledge bases, not all knowledge bases meet the criteria to be labeled as knowledge graphs.

To offer personalized diagnostic recommendations using gene expression profiles, it is important to obtain knowledge relevant to individual patient data. Huser et al. use the term ‘knowledge bases’ to describe resources that include information about the interpretation and implications of specific genomic findings [18]. They further mention that knowledge bases typically contain aggregated knowledge and no patient-level data [18].

In our case, the patient’s data that we analyse is their gene expression profile, while the knowledge base can encompass additional information related to genes, diseases, and associations between them. This knowledge can be accessed through knowledge bases such as CTD [19], GisGeNet [20], Gene Ontology [21] and Disease Ontology [22, 23].

Researchers are exploring new ways to use the knowledge represented by biomedical KBs to solve complex problems in the biomedical domain [24–27]. Bonner et al. [28] provide an overview of existing biomedical KBs. Biomedical KBs such as DisGeNet [20], Hetionet [29], BioKG [30], Bio2RDF [31, 32] and UniProt [33] provide prior biomedical knowledge which can be combined with patients’ clinical data for better model building in the health care domain.

Using DEGs, it is possible to measure changes in individual patients at the molecular level and identify the relevant biological processes triggered by those DEGs. Thus, DEGs can play an important role in disease diagnosis. However, a DEG can be involved in multiple biological processes [34, 35], and so be related to multiple different diseases in a KB. This makes it more challenging to perform personalised disease diagnosis based on DEGs, when there are thousands of diseases in the KB and the objective is to identify the most probable disease for a patient.

The existing biomedical KBs that we explored for experiments in this paper are not available with any quantitative association strength information. This leads to an implicit assumption that all associations are of equal significance or strength. For example, in biomedical KBs such as DisGeNet,^1^ if there is an association between a gene and a disease, the KB does not represent the quantitative strength of the association between the gene and the disease. As a result, all genes are simply represented as being linked to all associated diseases, but in reality, only a small group of genes are strongly associated to a particular disease, while many other genes are weakly associated to it. This can limit the usefulness of such KBs for identifying which diseases are most likely, given observed gene expression data.

Moreover, KBs are known to be generally quite incomplete. For example, more than 60% of the people in DBpedia and Freebase are missing their birthplaces [13, 36, 37]. Similarly, biomedical KBs also suffer from the problem of missing links. No existing KB has information of all possible diseases and related entities. For example, there are more than 10,000 rare diseases [38] and most of the biomedical KBs have between 2000 and 9000 diseases [39]. Missing links can be added based on the literature, as we will describe in “CTD knowledge base” section. Missing links can also be identified using KB embedding approaches [37, 40, 41], however, curated KB links are considered more reliable. KB embedding approaches such as TransE were found to perform poorly in biomedical link prediction (13.88% Hits@10) [42]. Challahan et al. [43] also noted that KB embedding and NLP based biomedical KBs are generally very noisy and should be used with caution. Therefore, we have curated some missing links for the KB that we use in this work; see “CTD knowledge base” section. We have also performed experiments with the publicly available DisGeNet KB, without adding or changing any of its links.

The long-established Cyc KB [44] also illustrates the challenges of KB incompleteness. Cyc KB is still reported to have gaps, despite being estimated to have accumulated over 900 person-years of work in its development [16]. It may require many more person-years to refine such existing KBs to incorporate quantitative link strength manually. Therefore, in this paper, we propose an alternative approach that can make use of existing KBs and assist with disease diagnosis tasks, when we do not know the quantitative link strength values between genes and diseases in the KB.

Our overall goal is to predict the disease that a patient is most likely to have at a particular time, by evaluating changes in their gene expression levels, with the help of a KB that represents thousands of diseases and their links to associated genes and other entities. Bharadhwaj et al. [45] worked on combining gene expression data with biomedical KBs, however, their approach is not suitable for longitudinal gene expression datasets, where subjects’ samples collected at different time-points play an important role. The novelty and advantage of our proposed approach are that it is suitable for longitudinal gene expression datasets and that it considers the time aspect for personalised disease diagnosis.

Our specific contributions are as follows:

1. We demonstrate how a patient’s Least Differentially Expressed Genes (LDEGs) along with Most Differentially Expressed Genes (MDEGs) can help in disease diagnosis in the presence of a KB. To the best of our knowledge, LDEGs have not previously been used for disease diagnosis in combination with KBs.
2. We show how KBs that do not include quantitative link strength information can be used to infer the strength of links in a patient-specific manner, using the patient’s gene expression profile.
3. We propose two new algorithms to combine patients’ time-series gene expression data with a KB. Both of the algorithms can assist with personalised disease diagnosis and can produce a short personalised ranked list of most likely diseases for each patient.

The rest of the paper is structured as follows. In “Description of existing ML algorithms” section, we briefly describe the existing machine learning (ML) algorithms. “Gene expression datasets” section describes the real-world gene expression datasets that we will use in this work. “Knowledge base” section describes the KBs used to perform experiments. In “Proposed algorithms” section, we explain our proposed algorithms. “Experimental design” section describes the experimental design. In “Results” section, we discuss and compare results in detail. Finally, we conclude in “Conclusions and future work” section.

## Description of existing ML algorithms

In “Results” section, we will compare the performance of the proposed algorithms with existing ML algorithms. These ML algorithms are described here.

*k*-Nearest Neighbour (*k*-NN) is an instance-based learning algorithm [46]. *k*-NN stores the training cases, and when presented with a new query case, it finds the set of *k* instances that have the lowest distance, according to some metric; these are termed the nearest neighbours. Then, the query case is assigned a class label based on the majority class of the *k* nearest neighbours [47]. We have used the Euclidean distance metric for our experiments. The optimum value of *k* is searched over the range of $k = 1$ to *n* with a step size of 2 (odd values such as $1, 3,\ldots, n$), where *n* represents the number of samples in the training set. We chose odd values for *k* to avoid ties.

Random Forests is an ensemble machine learning method. It is considered an efficient algorithm for the classification of gene expression data [48]. The Random Forest algorithm constructs an ensemble of many classification trees [49, 50]. Each classification tree is created by selecting a bootstrap sample from the whole training dataset and a random subset of attributes with size denoted $n_a$ is selected at each split. The optimum value of $n_a$ is searched over the range of 10 to *x* with a step size of 10, where *x* represents the square root of the total number of attributes (in this case, the total number of genes). The number of trees in the ensemble is denoted as $n_t$. We have used $n_t = 100$.

Support Vector Machine (SVM) works on the principle of finding the maximum margin separating hpyerplane. Assume that we have a training set of instance-label pairs $({\varvec{x}}_{i},y_{i}); \forall i \in \{1, 2,\ldots ,l\}$ where ${\varvec{x}}_{i} \in {\mathbb {R}}^{n}$ and ${\varvec{y}} \in \left\{ 1,-1\right\} ^{l}$, then the SVM [51–53] can be formulated and solved by the following optimization problem:

1 $$\begin{aligned}&\underset{{\varvec{w}},b,\varvec{\xi _{i}}}{\text {min}} & \frac{1}{2}{{\varvec{w}}^{T} {\varvec{w}}} + C \sum _{i=1}^{l} \xi _{i}, \\&\text {subject to} & y_{i}\left( {\varvec{w}}^{T}\phi \left( {\varvec{x}}_{i} \right) + b\right) \ge 1-\xi _{i }, \\ & &\xi _{i } \ge 0. \end{aligned}$$

Here ${\varvec{w}}$ is normal to the hyperplane, $\phi$ is a function that maps the data into a higher dimensional space, the parameter $C>0$ is the penalty parameter of the error term [53] and $\xi _{i} \forall i \in \{1, 2,\ldots ,l\}$ are positive slack variables [51].

Furthermore, $K(\varvec{x_{i},x_{j}}) = \phi (\varvec{x_{i}})^{T}\phi (\varvec{x_{j}})$ is called the kernel function [53]. The technique known as the kernel trick [54] can be used to translate the linear SVM algorithm into a kernelized version. After projecting data into a higher dimensional space, the SVM finds a maximal margin linear classifier, $f({\varvec{x}}) = sign({\varvec{w}}^{T}\phi ({\varvec{x}}))$ which can be solved using Eq. (1). There are four basic kernels that are frequently used: linear, polynomial, sigmoid, and RBF. We produced results using both Linear SVM and using SVM with RBF kernel.

For Linear SVM (linear kernel: $K(\varvec{x_{i},x_{j}}) = \varvec{x_{i}}^{T}\varvec{x_{j}}$), we did a search for best value of parameter *C* for a range of values from $2^{-5}$ to $2^{15}$ in multiples of 4.

We also used SVM with RBF kernel which is a non-linear kernel. We picked the RBF kernel, as recommended by Hsu et al. [53]. It has the following form:

$$\begin{aligned} \begin{array}{l} K\left( {\varvec{x}}_{i},{\varvec{x}}_{j} \right) = \exp \left( \frac{-\left\| {\varvec{x}}_{i}-{\varvec{x}}_{j} \right\| ^{2}}{2\sigma ^{2}} \right) ; \frac{1}{2\sigma ^{2}}> 0. \end{array} \end{aligned}$$

We performed a grid-search over the values of *C* and $\sigma$. The different pairs of $\left( C,\sigma \right)$ values are tried in the range of $(C=2^{-5},2^{-3},\ldots ,2^{15}; \sigma = 2^{-15}, 2^{-13},\ldots , 2^{3})$.

XGBoost (eXtreme Gradient Boosting) [55] is an ensemble learning algorithm that has been found to be an effective method for a wide range of machine learning tasks, including classification, regression, and ranking. XGBoost builds a set of decision trees iteratively, using a gradient boosting approach to minimize a user-specified loss function.

The key idea behind XGBoost is to iteratively add decision trees to the ensemble, with each new tree trained to correct the residual errors of the previous trees. In other words, XGBoost fits the model by adding new trees to the ensemble that improve the overall prediction accuracy, while penalizing trees that are too complex or overfit the data. We used the R implementation of the XGBoost library with its default gradient boosting tree model, called GBTree.^2^ The optimal values for XGBoost parameters were determined across the following ranges: *eta* (learning rate) from 0.1 to 1 with a step size of 0.1, $max\_depth$ (maximum depth of a tree) from 2 to 6, and *nround* (number of rounds in the gradient boosting process) from 10 to 100 with a step size of 10.

## Gene expression datasets

We have conducted experiments using four real-world gene expression datasets related to Respiratory Viral Infection (RVI). Dataset 1 is collected from 7 RVI Challenge studies, and is openly available on Gene Expression Omnibus (GEO).^3^ This dataset consists of 151 human volunteers who were healthy when they enrolled for the study. After enrolment, all subjects were inoculated with one of four viruses (H1N1, H3N2, HRV, RSV). Their blood samples were taken at pre-defined time-points, including before inoculation, thus delivering gene expression profiles from non-infected individuals as well as from infected ones [56]. Out of 151 subjects, 47 subjects samples failed quality control checks, so we exclude them from the study. For more information, see [57, 58].

Dataset 2 contains gene expression profiles of 133 adults whose samples are taken in three different seasons: Autumn, Winter and Spring. Baseline samples are taken at the time of enrolment of volunteers [59]. For each volunteer, samples are taken at up to seven time-points before, during, and after the occurrence of illness (influenza and other acute respiratory viral infections). This dataset is also accessible on GEO.^4^

Dataset 3, also on GEO,^5^ is collected from an influenza challenge trial in which 21 volunteers participated. Their samples are collected at baseline (healthy) and 4 different time-points after intranasal administration of wild-type A/California/2009 H1N1 virus [60]. Out of 21 subjects, 15 got infected and reported symptoms of illness. Three more subjects had some detectable amount of live virus shedding [60], however, their mapping to subject IDs are not available, therefore, we performed experiments with the data of the 15 subjects for whom reliable information is available.

Dataset 4 is also collected from an influenza trial which contains the gene expression profile of 22 subjects. All subjects were healthy at the time of enrollment and were aged 18–45 years [61]. All 22 subjects were inoculated with A/Wisconsin/67/2005 H3N2 influenza virus at a dose of 1 ml in a quarantine facility [61]. Gene expression data from peripheral blood was taken immediately before the viral inoculation and at 12, 24, and 48 h post-inoculation [61]. Dataset 4 is also accessible on GEO.^6^

## Knowledge base

We performed experiments using two KBs: DisGeNet KB [20] and CTD KB [19]. The following subsections provide a description of these KBs.

### DisGeNet knowledge base

The DisGeNet KB [20] is a publicly available collection of genes, diseases, and variants associated with human diseases. For sake of simplicity and for the requirement of the research work, we extracted a subset of the DisGeNet KB from the provided portal^7^ using the R package mentioned on the portal website. The extracted DisGeNet KB has 7 types of entities and 6 types of relations. The full RDF schema of DisGeNet KB is available on DisGeNet website.^8^ The 7 types of entities that our experimental DisGeNet KB includes are gene, disease, disease type, disease class, disease semantic type, protein class, and UniProt ID. A UniProt ID is linked with a gene, representing a specific protein encoded by that gene. UniProt IDs provide information about the gene that encodes a particular protein, including its gene symbol and chromosomal location, as well as the function and interactions of the protein through the UniProt KB [62]. We included UniProt IDs in our experimental DisGeNet KB so that it becomes easier for researchers to further investigate these relationships if they want to do so.

**Fig. 1:**
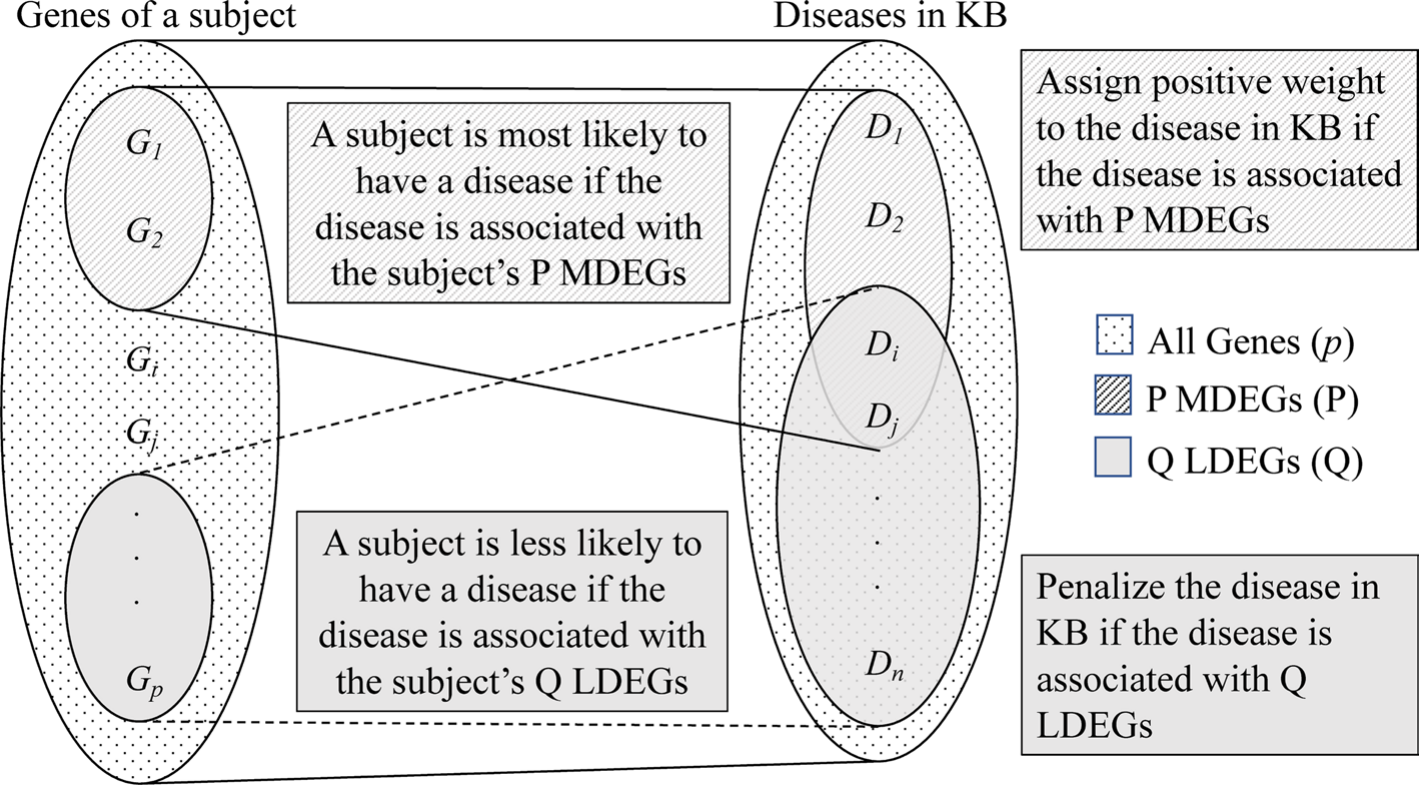
The novel idea based on which we designed both the proposed algorithms. MDEGs and LDEGs are the abbreviations for Most Differentially Expressed Genes and Least Differentially Expressed Genes respectively

### CTD knowledge base

CTD KB is described as a digital ecosystem that establishes connections between toxicological data pertaining to genes, diseases, chemicals, and phenotypes. [19, 63]. CTD KB^9^ has been extensively used in projects where the association between biomedical entities plays an important role [64, 65]. There are 11,622 genes which are common between the CTD KB and the experimental gene expression data set so we use only these. After pre-processing, the CTD KB has a total of 14,138,823 links between 11,622 genes and 6430 diseases.

We found that the CTD KB does not have RVI disease links so we added curated RVI links to it. To do this, we referred to five journal papers [56, 59, 66–68] to find information about which genes are associated with RVI. All relevant genes as identified in the journal papers were already present in the CTD KB, so we added 220 links from them to the new RVI disease in the KB. See Fig. S1 in Additional file 1 that plots the disease in-degree of the KB, which we define as the number of genes linked to each disease. The CTD KB we are using for our experiments also has 7 types of entities and 6 types of relations, because for those genes and diseases that are common between CTD and DisGeNet, we have added other entity types and relations in the CTD KB from DisGeNet KB.

## Proposed algorithms

Our approach to personalised disease diagnosis is inspired by the approach of recommender systems, where the goal is to provide a short ranked list of recommended items to a person, from a set of thousands of items, based on the person’s past preferences or profile. Here, we aim to provide a short ranked list of most likely diseases from the thousands in the KB, based on the person’s gene expression profile.

For that, we have developed two algorithms, LOADDx (Log-Odds based Assistant for Disease Diagnosis (Dx)) and SCADDx (SCore-based Assistant for Disease Diagnosis (Dx)). Both of the algorithms share the same basic novel idea of up-weighting disease scores based on *P* MDEGs, and down-weighting disease scores based on *Q* LDEGs, where the *P* MDEGs (Most Differentially Expressed Genes) are the top *P* ranked genes whose expression levels show a large difference between the healthy state (control) and the diseased state (target). Conversely, the *Q* LDEGs (Least Differentially Expressed Genes) are the bottom *Q* ranked genes whose expression levels show little or no difference between the two states. In effect, genes are sorted in descending order of their differential expression, and we select the top *P* and bottom *Q*. The idea is that, for a given person at a particular time *t*, if a significant number of MDEGs are associated with a particular disease in the KB, this provides evidence supporting that the person may have that disease, so the disease is up-weighted based on the identified MDEGs. Conversely, if a significant number of LDEGs are associated with a particular disease, this provides evidence against the person having that disease, so the disease is down-weighted. Then, the disease with the highest weight should be the most likely disease that the person may have at that time. Figure 1 illustrates the basic idea.

In order to test our hypothesis that the magnitude of most/least differentially expressed genes may be a useful signal in relating gene expression to disease diagnosis, we propose two algorithms: LOADDx does not use the magnitudes of MDEGs/LDEGs, whereas SCADDx does. Then, by testing whether SCADDx outperforms LOADDx, we will gain insight into whether this magnitude information is important.

### LOADDx algorithm

The LOADDx algorithm finds the changes in all genes’ expression levels ($\Delta G$) by subtracting a subject’s gene expression data at time = $t_{1}$ (healthy state) from their gene expression data at time = $t_{D}$, the time at which disease diagnosis has been requested (infected state or when infection is suspected). It selects the *P* MDEGs and *Q* LDEGs from the sorted list of all DEGs. Then, for each disease in the KB, it finds the number of common genes *CP* between those associated with disease $D_{i}$ and the *P* MDEGs from the gene expression data. It calculates the log-odds (*LP*) of disease $D_{i}$ from the *P* LDEGs and *CP* genes as follows:

2 $$\begin{aligned} LP=\ln (CP+1/(P+1-CP) ) \end{aligned}$$

**Algorithm 1:**
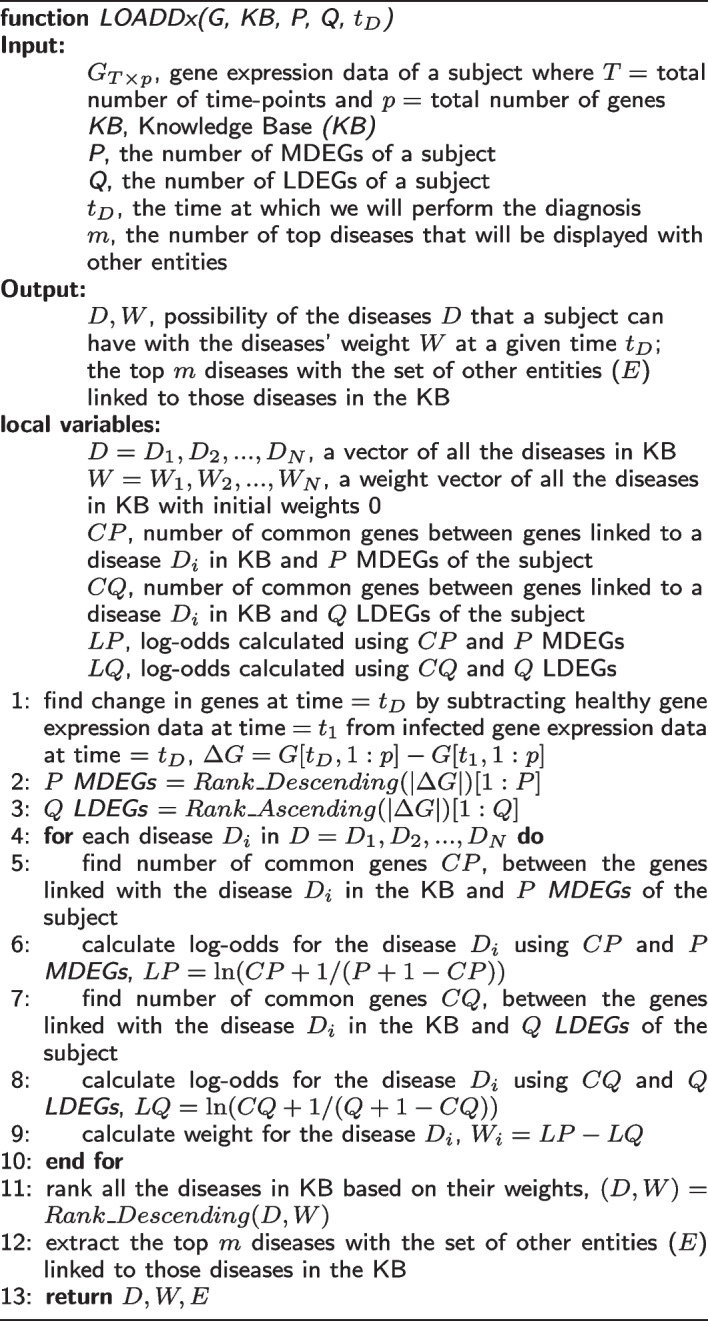
LOADDx Algorithm

**Algorithm 2:**
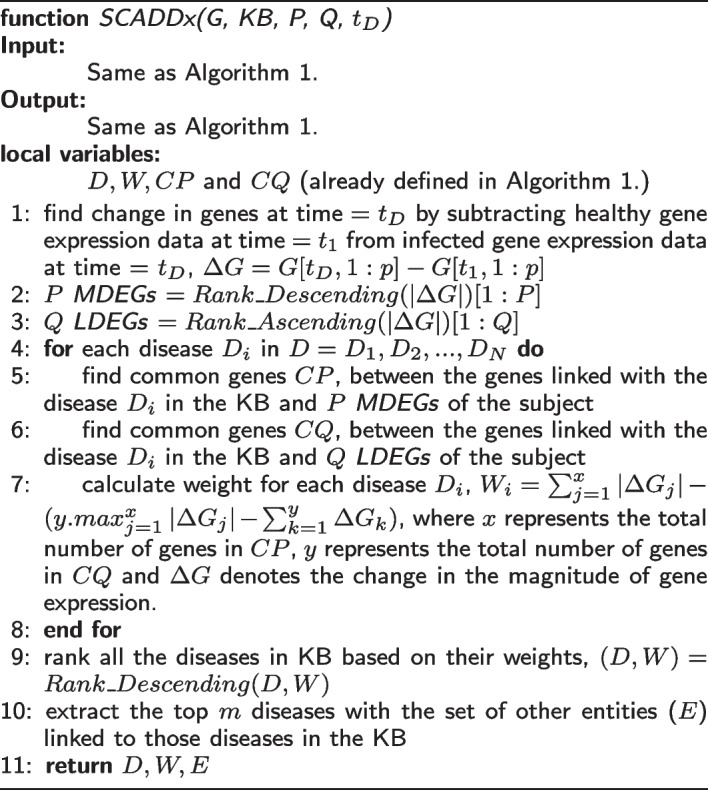
SCADDx Algorithm

Similarly, it finds the number of common genes *CQ* between those associated with disease $D_{i}$ and the *Q* LDEGs in the gene expression data. It calculates the log-odds (*LQ*) of disease $D_{i}$ from the *Q* LDEGs and *CQ* genes as follows:

3 $$\begin{aligned} LQ=\ln (CQ+1/(Q+1-CQ) ) \end{aligned}$$

Then, it calculates the weight $W_{i}$ for each disease $D_{i}$ using the following formula:

4 $$\begin{aligned} W_{i}= LP - LQ \end{aligned}$$

Finally, it ranks all the diseases in the KB in descending order based on their calculated weights/scores and extracts the top *m* diseases with the set of other entities (*E*) linked to those diseases in the KB.

### SCADDx algorithm

The SCADDx algorithm operates on the same basic idea as that of the LOADDx algorithm. The most fundamental difference between them is that, to calculate disease score, SCADDx makes use of the magnitudes of the *P* MDEGs and *Q* LDEGs, whereas LOADDx does not.

SCADDx selects *P* MDEGs and *Q* LDEGs from the list of all DEGs. Then, for each disease in the KB, it finds the common genes *CP* and *CQ* between those associated with disease $D_{i}$ and the *P* MDEGs and *Q* LDEGs respectively in the gene expression data. It calculates the weight $W_{i}$ for each disease $D_{i}$ using the following formula:

5 $$\begin{aligned} W_{i} = \sum _{j=1}^{x} \left| \Delta G_{j}\right| - ( y.max_{j=1}^{x}\left| \Delta G_{j} \right| - \sum _{k=1}^{y} \Delta G_{k} ) \end{aligned}$$

where *x* represents the total number of genes in *CP*, *y* represents the total number of genes in *CQ* and $\Delta G$ denotes the change in the magnitude of gene expression. Finally, it ranks all the diseases in the KB in descending order based on their calculated weights and extracts the top *m* diseases with the set of other entities (*E*) linked to those diseases in the KB.

To compute the probabilities of top-ranked *m* diseases from their weight scores, we use the softmax function, $f(w_{i}) = e^{w_{i}}/\sum _{j=1}^{m} e^{w_{j}}$, where, $w_{i}$ = weight of $i^{th}$ disease, *m* = number of diseases, $i = 1,\ldots , m$, and $f(w_{i})$ represents the probability [69].

## Experimental design

We design our experiments in a way such that it is possible to enable personalized disease diagnosis at an early stage of infection. We perform experiments by combining a KB with the patients’ gene expression data collected at an early time-point. Here, an early time-point means within day 3 (72 h) of exposure to a virus. For each subject, we consider gene expression data collected at two time-points. The first time-point is called the reference sample and the second time point is called the target sample. The reference sample is collected at time $t_{1}$ = 0 h, before the patient has the disease. Target samples are collected at time $t_{D}$ = day 2 or day 3, based on the availability of data, at which time subjects might or might not be exhibiting signs of infection. For all subjects in all datasets, reference samples are available at time $t_{1}$ = 0 h, however, target samples are not available at the same time for all subjects. For Dataset 1, we have target samples available between time $t_{D}$ = 60 to 72 h. For Dataset 2, we have target samples available at time $t_{D}$ = day 2. For Dataset 3 and Dataset 4, we have target samples available at time $t_{D}$ = 72 h and $t_{D}$ = 48 h respectively.

We test our proposed algorithms on four real-world gene expression datasets of RVI disease that are described in “Gene expression datasets” section. The datasets have a *true class* label indicating whether a subject has a respiratory viral infection or not (see Table 1). We use the *true class* label (actual disease) and *predicted class* label (predicted disease) to compute the accuracy of disease predictions. The formula to compute accuracy is: $Accuracy = (TP + TN)/(TP + TN + FP + FN)$, *where TP = True Positive, TN = True Negative, FP = False Positive, and FN = False Negative* [70]. For each patient, the *predicted class* label is obtained by comparing the actual disease with the top *n* predicted diseases, for values of *n* = 1, 2, 3, 4, 5 or 10. If there is a match found between the predicted top *n* diseases and the actual disease, then this is assigned as the *predicted class* label. For example, if the actual disease is *RVI*, and the algorithm includes *RVI* in its top *n* predicted diseases, the *predicted class* label is set to *RVI*, otherwise it is set to *Not RVI*. Influenza is a respiratory viral infection that belongs to the class of respiratory tract infections or diseases. As a result, our proposed algorithms have the ability to identify respiratory viral infections or diseases if the KB contains any of these terms: Influenza or Respiratory Viral Infection, or Respiratory Tract Disease.

Because of the small size of the datasets, it would not be practical to use k-fold CV, even for small values of k such as 5. Therefore, we employed two alternative validation approaches to compare the performance of SCADDx and LOADDx with existing ML algorithms. The first approach is single internal validation set approach (see Tables 5, 6) and the second is Leave-One-Out Cross-Validation (LOOCV) approach (see Table 7). For a fair comparison, the existing ML models are also trained on the same time points for which the proposed algorithms are trained.

For the single internal validation set approach, all the datasets are divided into training, validation, and test sets with a ratio 50:25:25. We used random stratified sampling while splitting the datasets. The model parameters are selected based on the performance of the validation set. The power of the t-test increases as we increase the number of test sets, therefore, we divided the test set of Dataset 1 and Dataset 2 further into two parts (Testset 1a, 1b, 2a, and 2b). Thus, we have 6 test sets in total as shown in the tables in the “Results” section. We cannot divide test sets of Dataset 3 and Dataset 4 further as they are small.

LOOCV is considered an efficient way to evaluate performance when the number of samples is very small [56, 71]. Therefore, we also performed evaluations using the LOOCV approach. To conduct LOOCV, the data of each subject is held out one at a time as a test case, while the data of other subjects are used for training. LOOCV ensures there is no risk of a lucky split since each patient’s data serves as the validation set in each iteration, with all other data points acting as the training set. This process is repeated for each data point, and the results are then averaged to evaluate the model’s performance [71]. In our study, LOOCV was chosen to address the challenges posed by a limited number of samples. For LOOCV, both Dataset 1 and Dataset 2 are split into two equal parts, creating two independent datasets that we refer to as Datasets 1a, 1b, 2a and 2b. Each split consists of 50% of the data from its respective dataset. Comparative analysis with detailed results is presented in the next section.

## Results

In this section, we present the results of LOADDx and SCADDx using different parameter settings and comparative analysis using different values of *n*. We also analyse the performance of both algorithms on respiratory viral infections generally, as well their performance on specific virus types/subtypes in the datasets that can cause respiratory viral infections. Finally, we also compare the performance of the proposed algorithms with existing ML algorithms.

**Table 1:** Sample of results for the first 5 subjects of Testset 1a using SCADDx on CTD KB. Showing top 5 diseases for each subject with most affected 5 genes of the subject

Subject ID	Top 5 genes (abs($\Delta$G))	Disease name	Disease score	Disease probability (Softmax) (%)	Predicted class label	True class label
1	PDIA3 (0.22) RALGDS (0.21) TNKS2 (0.21) NCKAP1L (0.21) ANXA6 (0.20)	Dysentery, Bacillary	0.73	21.81	Not RVI	Not RVI
		Colonic Diseases, Functional	0.64	19.99		
		Esophageal Motility Disorders	0.64	19.99		
		Hypochondriasis	0.61	19.35		
		Encephalitis, Herpes Simplex	0.58	18.86		
2	APBB1IP (0.33) HBB (0.29) TAGLN2 (0.27) USP34 (0.216) FAM106A (0.25)	Subdural Effusion	0.65	23.33	Not RVI	RVI
		Dysentery, Bacillary	0.65	23.33		
		Penile Neoplasms	0.43	18.77		
		Hepatitis, Viral, Animal	0.35	17.36		
		Antley-Bixler Syndrome Phenotype	0.34	17.21		
3	IFI27 (0.44) IFI44L (0.30) SPATS2L (0.29) IFI44 (0.29) RSAD2 (0.29)	Respiratory Viral Infection	9.65	92.66	RVI	RVI
		Failure to Thrive	5.82	2.02		
		Paraparesis, Tropical Spastic	5.69	1.77		
		Mitochondrial myopathy with lactic acidosis	5.69	1.77		
		Retroviridae Infections	5.69	1.77		
4	CSTA (0.16) KLRB1 (0.13) NDUFA1 (0.12) ATP5F1 (0.12) RPL36AP37 (0.12)	Extensively Drug-Resistant Tuberculosis	0.39	22.7	Not RVI	Not RVI
		Phantom Limb	0.23	19.4		
		Trochlear Nerve Diseases	0.23	19.4		
		Alexander Disease	0.22	19.3		
		Epilepsy, Benign Neonatal	0.22	19.3		
5	DMXL1 (0.22) BMI1 (0.20) MYBL1 (0.19) ZBTB11 (0.17) PLEKHF2 (0.17)	Osteosclerosis	0.54	20.85	Not RVI	Not RVI
		Echolalia	0.53	20.78		
		Contracture	0.50	20.03		
		Esophageal Stenosis	0.49	19.81		
		Appendiceal Neoplasms	0.42	18.52		

Parameter values: *P* = 100, *Q* = 175, *m* = 5, time $t_{D} \simeq 60$ hours

### Comparison of LOADDx with SCADDx

For each subject, both SCADDx and LOADDx produce a ranked list of the top *n* predicted diseases with their weights and probabilities (see Table 1 and Tables S1–S12 in Additional file 1). These tables present the results obtained using the single internal validation set approach, as explained in “Experimental design” section. For hyperparameter optimization of SCADDx and LOADDx, we conducted a grid search over *P* and *Q* in the range of 25 to 300 with a step size of 25. Table 1 shows the top 5 diseases predicted by the SCADDx algorithm for first 5 subjects for Testset 1a. Please see Additional file 1 for full results on all the datasets using both algorithms. Table 1 also shows, for each subject, the top 5 most affected genes, and the changes in expression values of these genes. The subjects with the largest changes in gene expression have the highest disease scores in SCADDx (see Table 1, Subject 3). These can indicate severe cases of infection, so such subjects should be handled carefully. From Table 1, it can be seen that our SCADDx algorithm can produce a short personalised ranked list of most likely diseases for each patient, which can help health care professionals in their decision-making.

**Table 2:** Comparison between SCADDx and LOADDx using CTD KB

Datasets	Algorithm	Parameter values	Accuracy n@1 (%)	Accuracy n@2 (%)	Accuracy n@3 (%)	Accuracy n@4 (%)	Accuracy n@5 (%)	Accuracy n@10 (%)
Dataset 1 Testset 1a (GSE73072)	SCADDx	P = 200 Q = 200	76.92	76.92	76.92	76.92	76.92	76.92
	LOADDx	P = 200 Q = 200	69.23	69.23	69.23	69.23	69.23	69.23
Dataset 1 Testset 1b (GSE73072)	SCADDx	P = 200 Q = 200	84.62	84.62	84.62	84.62	84.62	76.92
	LOADDx	P = 200 Q = 200	76.92	76.92	76.92	76.92	76.92	76.92
Dataset 2 Testset 2a (GSE68310)	SCADDx	P = 200 Q = 200	100	100	100	100	100	100
	LOADDx	P = 200 Q = 200	75	75	87.5	87.5	87.5	100
Dataset 2 Testset 2b (GSE68310)	SCADDx	P = 200 Q = 200	86.66	86.66	86.66	86.66	86.66	93.33
	LOADDx	P = 200 Q = 200	86.66	86.66	86.66	86.66	86.66	86.66
Dataset 3 (GSE90732)	SCADDx	P = 200 Q = 200	100	100	100	100	100	100
	LOADDx	P = 200 Q = 200	100	100	100	100	100	100
Dataset 4 (GSE61754)	SCADDx	P = 200 Q = 200	85.71	85.71	85.71	85.71	85.71	85.71
	LOADDx	P = 200 Q = 200	85.71	85.71	85.71	85.71	85.71	85.71

Parameter values: *P* = *Q* = 200 genes for all the four datasets

**Fig. 2:**
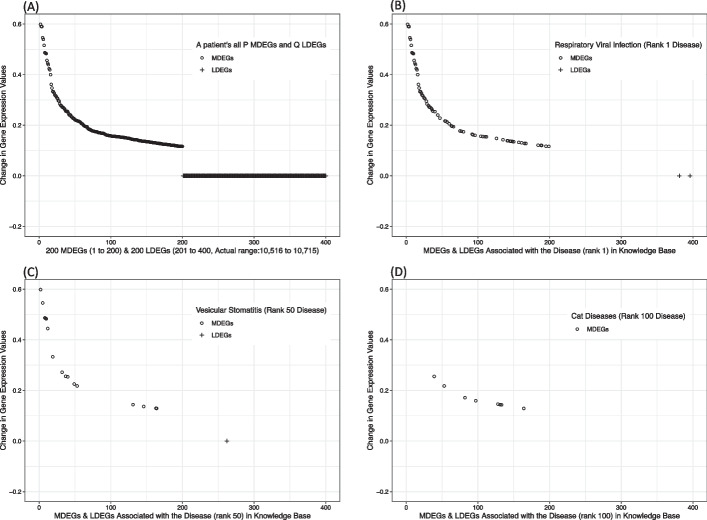
A visualisation of results of SCADDx using CTD KB for a single subject and a single dataset (first subject of Dataset 2). **A** shows change in gene expression value of all the *P* MDEGs and *Q* LDEGs of subject 1. **B** shows only those genes (MDEGs and LDEGs) of subject 1 which are associated with the disease in KB which has been assigned rank 1 by SCADDx. **C**, and **D** show only those genes (MDEGs and LDEGs) of subject 1 which are associated with the diseases in KB which have been assigned ranks 50 and 100 respectively by SCADDx

**Table 3:** Comparison between SCADDx and LOADDx using CTD KB considering best parameter values (*P* & *Q*) for all the four datasets

Datasets	Algorithm	Parameter values	Accuracy n@1 (%)	Accuracy n@2 (%)	Accuracy n@3 (%)	Accuracy n@4 (%)	Accuracy n@5 (%)	Accuracy n@10 (%)
Dataset 1 Testset 1a (GSE73072)	SCADDx	P = 100 Q = 175	76.92	76.92	76.92	76.92	76.92	84.62
	LOADDx	P = 25 Q = 225	69.23	69.23	69.23	69.23	69.23	69.23
Dataset 1 Testset 1b (GSE73072)	SCADDx	P = 100 Q = 175	84.62	76.92	76.92	76.92	76.92	76.92
	LOADDx	P = 25 Q = 225	84.62	84.62	84.62	84.62	84.62	84.62
Dataset 2 Testset 2a (GSE68310)	SCADDx	P = 150 Q = 300	100	100	100	100	100	100
	LOADDx	P = 50 Q = 300	75	81.25	87.5	87.5	93.75	100
Dataset 2 Testset 2b (GSE68310)	SCADDx	P = 150 Q = 300	86.66	93.33	93.33	93.33	93.33	100
	LOADDx	P = 50 Q = 300	80	80	80	80	80	86.66
Dataset 3 (GSE90732)	SCADDx	P = 25 Q = 25	100	100	100	100	100	100
	LOADDx	P = 25 Q = 25	75	100	100	100	100	100
Dataset 4 (GSE61754)	SCADDx	P = 25 Q = 25	85.71	85.71	85.71	85.71	85.71	85.71
	LOADDx	P = 25 Q = 50	85.71	85.71	85.71	85.71	85.71	85.71

**Table 4:** Virus-wise comparison between SCADDx and LOADDx using CTD KB

Dataset	Algorithm (Parameters)	Virus	Subjects ($S^{+}/S$)	Accuracy n@1 (%)	Accuracy n@2 (%)	Accuracy n@3 (%)	Accuracy n@4 (%)	Accuracy n@5 (%)	Accuracy n@10 (%)
Dataset 1 Testset 1a (GSE73072)	SCADDx P = 100 Q = 175	H1N1 virus	1/2	50	50	50	50	50	50
		H3N2 virus	1/3	100	100	100	100	100	100
		HRV virus	4/5	100	100	100	100	100	100
		RSV virus	2/3	33.33	33.33	33.33	33.33	33.33	66.66
	LOADDx P = 25 Q = 225	H1N1 virus	1/2	50	50	50	50	50	50
		H3N2 virus	1/3	100	100	100	100	100	100
		HRV virus	4/5	80	80	80	80	80	80
		RSV virus	2/3	33.33	33.33	33.33	33.33	33.33	33.33
Dataset 1 Testset 1b (GSE73072)	SCADDx P = 100 Q = 175	H1N1 virus	2/3	100	100	100	100	100	100
		H3N2 virus	3/4	75	75	75	75	75	75
		HRV virus	2/3	100	100	100	100	100	100
		RSV virus	1/3	66.66	33.33	33.33	33.33	33.33	33.33
	LOADDx P = 25 Q = 225	H1N1 virus	2/3	100	100	100	100	100	100
		H3N2 virus	3/4	75	75	75	75	75	75
		HRV virus	2/3	100	100	100	100	100	100
		RSV virus	1/3	66.66	66.66	66.66	66.66	66.66	66.66
Dataset 2 Testset 2a (GSE68310)	SCADDx P = 150 Q = 300	Influenza A virus	7/7	100	100	100	100	100	100
		Other viruses	3/3	100	100	100	100	100	100
		HRV virus	5/5	100	100	100	100	100	100
		Infected but no virus subtype detected	1/1	100	100	100	100	100	100
	LOADDx P = 50 Q = 300	Influenza A virus	7/7	85.71	85.71	100	100	100	100
		Other viruses	3/3	66.66	66.66	66.66	66.66	100	100
		HRV virus	5/5	80	100	100	100	100	100
		Infected but no virus subtype detected	1/1	0	0	0	0	0	100
Dataset 2 Testset 2b (GSE68310)	SCADDx P = 150 Q = 300	Influenza A virus	5/5	100	100	100	100	100	100
		Other viruses	5/5	100	100	100	100	100	100
		HRV virus	1/1	0	0	0	0	0	100
		Infected but no virus subtype detected	4/4	75	100	100	100	100	100
	LOADDx P = 50 Q = 300	Influenza A virus	5/5	80	80	80	80	80	100
		Other viruses	5/5	100	100	100	100	100	100
		HRV virus	1/1	0	0	0	0	0	0
		Infected but no virus subtype detected	4/4	75	75	75	75	75	75
Dataset 3 (GSE90732)	SCADDx P = 25 Q = 25	H1N1 virus	4/4	100	100	100	100	100	100
	LOADDx P = 25 Q = 25	H1N1 virus	4/4	75	100	100	100	100	100
Dataset 4 (GSE61754)	SCADDx P = 25 Q = 25	H3N2 virus	4/7	85.71	85.71	85.71	85.71	85.71	85.71
	LOADDx P = 25 Q = 50	H3N2 virus	4/7	85.71	85.71	85.71	85.71	85.71	85.71

$S^{+}$ denotes the number of infected subjects and *S* denotes the number of total subjects in the testset in that virus category

To compare the performance of proposed algorithms, we compute the accuracy of each algorithm based on whether the correct disease is in the top *n* predicted diseases. Table 2 presents a comparative analysis between LOADDx and SCADDx at different values of *n*, keeping the parameter values fixed ($P = 200, Q = 200$). SCADDx achieves a median accuracy of 89.52% (average accuracy = 88.81%), whereas LOADDx achieves a median accuracy of 86.19% (average accuracy = 86.42%), when *n* = 10 in both cases considering all datasets (see Table 2). SCADDx performs as well as or better than LOADDx on all four datasets for all values of *n*. In almost cases, accuracy scores are higher for higher values of *n*. This is to be expected, since it is more likely that the correct answer would be among the top 3 ranked diseases than the top 2, for example. However, as we increase *n*, there is an increased risk of false positives. We can see this in the SCADDx result for Testset 1b, when *n* is increased from 5 to 10 (see Table 2). Therefore, we suggest that the number of predicted diseases (*n*) should be kept low (from 1 to 10). In any case, a short list of predicted diseases would be more useful for the user.

Table 3 presents a comparative analysis between LOADDx and SCADDx on different values of *n*, when performing a grid search over *P* and *Q* in the range of 25 to 300 with a step size of 25. The reported results were obtained by employing the single internal validation set approach, as explained in “Experimental design” section. The median accuracy of SCADDx is 92.86% (average accuracy = 91.21%), whereas for LOADDx the median accuracy is 86.19% (average accuracy = 87.70%) when *n* = 10 (see Table 3). Again, SCADDx performs better than LOADDx. The results of the grid search suggest that in most of the cases, both algorithms achieved their best accuracies when $Q > P$ (see Table 3). When $Q > P$, the algorithms down-weight those diseases that are linked to LDEGs (*Q*) in the KB, since having a larger number of LDEGs associated with a disease provides stronger evidence against the person having that disease. By down-weighing the diseases that a person is less likely to have, better accuracy is achieved.

There are many virus types/subtypes that can cause RVI disease. Therefore, we have performed what we term a virus-wise performance analysis, to analyse how well the proposed algorithms work on different viruses. The datasets have an entry named *virus* that provides the information about the type/subtype of virus that caused each subject’s infection (see Table 4). For example, Dataset 1 contains 4 specific viruses, as shown in Table 4. Please refer to Additional file 1 (see Table S13) for the information of all virus types/subtypes covered in all the datasets. We categorized all subjects into 7 general virus groups: H1N1, H3N2, HRV, RSV, Influenza A, Other viruses and Infected but no virus subtype detected (see Table 4).

**Table 5:** Comparing the performance of SCADDx and LOADDx with existing machine learning algorithms using the single internal validation set approach ($n=10$ for SCADDx and LOADDx)

Algorithm	Dataset 1			Dataset 2			Dataset 3		Dataset 4		Average accuracy (testsets) (%)
	Parameters	Accuracy		Parameters	Accuracy		Parameters	Accuracy	Parameters	Accuracy	
		Testset 1a (%)	Testset 1b (%)		Testset 2a (%)	Testset 2b (%)		Testset 3 (%)		Testset 4 (%)	
LOADDx (CTD KB)	P = 25, Q = 225	69.23	84.62	P = 50, Q = 300	100	86.66	P = 25, Q = 25	100	P = 25, Q = 50	85.71	${\textbf {*87.70}}$
SCADDx (CTD KB)	P = 100, Q = 175	84.62	76.92	P = 150, Q = 300	100	100	P = 25, Q = 25	100	P = 25, Q = 25	85.71	${\textbf {*91.21}}$
LOADDx (DisGeNet KB)	P = 275, Q = 50	84.62	92.31	P = 100, Q = 75	100	93.33	P = 25, Q = 25	100	P = 25, Q = 25	85.71	${\textbf {*92.66}}$
SCADDx (DisGeNet KB)	P = 300, Q = 100	84.62	84.62	P = 75, Q = 100	100	93.33	P = 25, Q = 25	100	P = 25, Q = 25	85.71	${\textbf {*91.38}}$
*k*-NN	K = 11	46.15	61.54	K = 13	87.5	93.33	K=1	75%	K=7	57.14	70.11
Random Forest	$n_{a}$ = 100, $n_{t}$ = 100	76.92	76.92	$n_{a}$ = 100, $n_{t}$ = 100	100	93.33	$n_{a}$ = 90, $n_{t}$ = 100	100	$n_{a}$ = 50, $n_{t}$ = 100	71.43	86.43
Linear SVM	C = $2^{-5}$	76.92	61.54	C = $2^{-5}$	100	93.33	C = $2^{-5}$	100	C = $2^{-5}$	71.43	83.87
SVM with RBF Kernel	$\sigma$ = $2^{-15}$, C = $2^{3}$	76.92	61.54	$\sigma$ = $2^{-15}$, C = $2^{0}$	75	86.67	$\sigma$ = $2^{-7}$, C = $2^{-1}$	100	$\sigma$ = $2^{3}$, C = $2^{0}$	42.86	73.83
XGBoost (GBTree)	*eta* = 0.3, $max\_depth$ = 2 *nround* = 30	76.92	76.92	*eta* = 0.3, $max\_depth$ = 2 *nround* = 20	100	93.33	*eta* = 0.8, $max\_depth$ = 2 *nround* = 10	100	*eta* = 0.1, $max\_depth$ = 2 *nround* = 90	71.43	86.43

Results in bold denote that they are statistically significant based on the performed t-test

A single asterisk denotes *p* value < 0.05

**Table 6:** Comparing the performance of SCADDx and LOADDx with existing machine learning algorithms using the single internal validation set approach ($n=1$ for SCADDx and LOADDx)

Algorithm	Dataset 1			Dataset 2			Dataset 3		Dataset 4		Average accuracy (testsets) (%)
	Parameters	Accuracy		Parameters	Accuracy		Parameters	Accuracy	Parameters	Accuracy	
		Testset 1a (%)	Testset 1b (%)		Testset 2a (%)	Testset 2b (%)		Testset 3 (%)		Testset 4 (%)	
LOADDx (CTD KB)	P = 25, Q = 225	69.23	84.62	P = 50, Q = 300	75	80	P = 25, Q = 25	75	P = 25, Q = 50	85.71	78.26
SCADDx (CTD KB)	P = 100, Q = 175	76.92	84.62	P = 150, Q = 300	100	86.66	P = 25, Q = 25	100	P = 25, Q = 25	85.71	${\textbf {*88.99}}$
LOADDx (DisGeNet KB)	P = 275, Q = 50	76.92	92.31	P = 100, Q = 75	93.75	93.33	P = 25, Q = 25	100	P = 25, Q = 25	85.71	${\textbf {*90.34}}$
SCADDx (DisGeNet KB)	P = 300, Q = 100	76.92	92.31	P = 75, Q = 100	100	93.33	P = 25, Q = 25	100	P = 25, Q = 25	85.71	${\textbf {*91.38}}$
*k*-NN	K = 11	46.15	61.54	K = 13	87.5	93.33	K=1	75	K=7	57.14	70.11
Random Forest	$n_{a}$ = 100, $n_{t}$ = 100	76.92	76.92	$n_{a}$ = 100, $n_{t}$ = 100	100	93.33	$n_{a}$ = 90, $n_{t}$ = 100	100	$n_{a}$ = 50, $n_{t}$ = 100	71.43	86.43
Linear SVM	C = $2^{-5}$	76.92	61.54	C = $2^{-5}$	100	93.33	C = $2^{-5}$	100	C = $2^{-5}$	71.43	83.87
SVM with RBF Kernel	$\sigma$ = $2^{-15}$, C = $2^{3}$	76.92	61.54	$\sigma$ = $2^{-15}$, C = $2^{0}$	75	86.67	$\sigma$ = $2^{-7}$, C = $2^{-1}$	100	$\sigma$ = $2^{3}$, C = $2^{0}$	42.86	73.83
XGBoost (GBTree)	*eta* = 0.3, $max\_depth$ = 2 *nround* = 30	76.92	76.92	*eta* = 0.3, $max\_depth$ = 2 *nround* = 20	100	93.33	*eta* = 0.8, $max\_depth$ = 2 *nround* = 10	100	*eta* = 0.1, $max\_depth$ = 2 *nround* = 90	71.43	86.43

Results in bold denote that they are statistically significant based on the performed t-test

A single asterisk denotes *p* value < 0.05

Table 4 represents virus-wise performance analysis on all the testsets. These results were obtained by using the single internal validation set approach, as explained in “Experimental design” section. Table 4 shows that SCADDx is able to achieve 100% accuracy in the case of Influenza A virus for all the testsets for all values of *n*. SCADDx is also able to achieve 100% accuracy in case of H3N2 and HRV virus on Testset 1a, H1N1 and HRV virus on Testset 1b, all virus types on Testset 2a, Influenza A and other viruses types on Testset 2b, and H1N1 virus on Dataset 3 for all values of *n*. This illustrates that the strengths of relationships between KB entities play an important role, and shows that the novel idea on which SCADDx is based has the potential to achieve up to 100% accuracy (see Tables 4, 5). Based on the virus-wise comparative analysis, it can be observed that SCADDx again performs better than LOADDx. The main reason why SCADDx tends to outperform LOADDx is that it makes use of the magnitudes of MDEGs/LDEGs, whereas LOADDx does not. We conclude that this magnitude information is important, and that SCADDx is successfully able to exploit this information to infer gene-disease link strengths in a patient-specific fashion, and incorporate this information in disease probability estimates.

Both of the algorithms are able to perform well in case of H1N1, H3N2 and HRV viruses. However, they do not perform so well in case of the RSV virus (see Table 4). We examined the datasets more deeply to understand why, and concluded that the KB does not have sufficient information about genes associated with RSV virus. It has information about only 30 genes that are related to the RSV virus - a case of KB incompleteness. This could be addressed in future work by conducting further KB curation work to add more links.

Figure 2 provides a visualisation of results for a single subject and a single dataset (first subject of Dataset 2). In Fig. 2A, we plot the most and least differentially expressed genes (MDEGs & LDEGs) of a subject. From Fig. 2B–D, we plot the subject’s MDEGs and LDEGs associated with the disease in the KB, with the disease rank predicted by SCADDx. For example, as can be seen in Fig. 2B, there are many genes which are highly expressed and linked with RVI disease (the top-ranked disease), whereas the lower-ranked diseases have relatively fewer highly-expressed genes linked with those diseases in the KB. As would be expected, the larger the number of MDEGs associated with a disease, the higher the chances are of having that disease. The trend from Fig. 2B–D shows that as we move from the Rank 1 disease to the Rank 100 disease, the number of associated MDEGs drops significantly. Also, the larger the number of LDEGs associated with a disease, the lower the chances are of having that disease. Figure S2 in Additional file 1 shows that as we move from the Rank 1 disease to the Rank 6000 disease, the number of associated LDEGs increases significantly. These trends provide evidence in support of the disease rank predicted by SCADDx.

Based on these observations, we conclude that there are three main contributing factors that influence which disease will get a high rank. Firstly, a significantly large number of MDEGs should be associated with the disease. Secondly, a low number of LDEGs should be associated with it. Thirdly, among the associated MDEGs, the change in gene expression should be larger in comparison to the MDEGs associated with other diseases. If a very large number of LDEGs of a patient is associated with a disease in KB, then that disease should never get a higher rank.

### Comparison with existing ML algorithms

We also compared LOADDx and SCADDx with a number of existing machine learning algorithms (see Tables 5, 6, 7). The machine learning algorithms applied are *k*-NN, Random Forest, XGBoost, Linear SVM, and SVM with RBF kernel.

To compare the performance with existing machine learning algorithms, we applied two validation approaches. The first approach is the single internal validation set approach (see Tables 5, 6) and the second is the LOOCV approach (see Table 7) as explained in “Experimental design” section. The aim of applying existing machine learning algorithms is to determine a baseline performance that can be obtained on these datasets. The performance of LOADDx and SCADDx can then be assessed through comparison.

Table 5 shows results obtained using the single internal validation set approach. This table represents the results of SCADDx and LOADDx using both CTD and DisGeNet KBs, with the value of *n* set to 10. For the optimal parameter selection of both SCADDx and LOADDx, we conducted a grid search over *P* and *Q* in the range of 25 to 300 with a step size of 25. Refer to “Description of existing ML algorithms” section for the criteria used in selecting hyperparameters for the existing machine learning algorithms. Single internal validation set results show that SCADDx and LOADDx are able to detect the infection with up to 100% accuracy in the case of Dataset 2 and Dataset 3 (see Table 5). Overall, SCADDx and LOADDx are able to detect the infection within 72 h of infection with an average accuracy of 91.21% and 87.70% using the CTD KB, and 91.38% and 92.66% using the DisGeNet KB, respectively, considering all four datasets. In contrast, Random Forest and XGBoost, which performed best among the existing machine learning algorithms, can detect the infection with only an average accuracy of 86.43%.

**Table 7:** Comparing the performance of LOADDx and SCADDx with the performance of existing machine learning algorithms using the LOOCV approach ($n=10$ for SCADDx and LOADDx)

Algorithm	Mean accuracy (LOOCV)						Average accuracy (datasets) (%)
	Dataset 1a (%)	Dataset 1b (%)	Dataset 2a (%)	Dataset 2b (%)	Dataset 3 (%)	Dataset 4 (%)	
LOADDx (CTD KB)	82.69	75	90.16	91.80	93.33	72.73	${\textbf {**84.29}}$
SCADDx (CTD KB)	80.77	80.77	96.72	95.08	93.33	72.73	${\textbf {**86.57}}$
LOADDx (DisGeNet KB)	82.69	78.85	96.72	95.08	93.33	72.73	${\textbf {**86.57}}$
SCADDx (DisGeNet KB)	84.62	76.92	96.72	93.44	93.33	72.73	${\textbf {**86.29}}$
*k*-NN	48.08	51.92	81.97	80.32	80	40.90	63.87
Random Forest	80.77	67.31	90.16	86.88	86.66	63.64	79.24
Linear SVM	73.08	75	90.16	90.16	73.33	59.09	76.80
SVM with RBF Kernel	76.92	65.38	90.16	90.16	73.33	59.09	75.84
XGBoost (GBTree)	80.77	69.23	91.80	88.52	86.66	54.54	78.59

Results in bold denote that they are statistically significant based on the performed t-test

A single asterisk denotes *p* value < 0.05 and a double asterisk denotes *p* value < 0.01

We also performed a paired t-test using the test set accuracy of all the four datasets. The t-test results on Table 5 show that SCADDx performs significantly better than three of the existing ML algorithms: *k*-NN; Linear SVM; and SVM with RBF Kernel. LOADDx performs significantly better than two: *k*-NN; and SVM with RBF Kernel. On average, SCADDx and LOADDx match or outperform the existing algorithms.

**Table 8:** Results of Gene Set Enrichment Analysis performed over the most important 16 genes that are common across all four gene expression datasets used in this study

Gene set for GSEA	Disease ontology ID	Disease or term	*p* Value	Odds ratio
RSAD2, IFI44L, RPS4Y1, IFI44, HERC5, ISG15, OAS3, IFIT3, OASL, SPATS2L, CCL8, OAS1, CCL2, OAS2, CXCL10, IFITM3	DOID:9001488	Human Influenza	7.72E-32	1747.6881
	DOID:8469	Influenza	7.86E−31	1464.2384
	DOID:9001499	Orthomyxoviridae Infections	1.06E−30	1431.0526
	DOID:9008680	Respiratory Tract Infections	2.75E−22	338.23645
	DOID:9002150	RNA Virus Infections	1.46E−17	224.25569
	DOID:934	Viral infectious disease	1.41E−16	190.4361
	DOID:0050117	Disease by infectious agent	6.66E−15	143.48608
	DOID:1579	Respiratory system disease	1.91E−12	93.22419
	DOID:0080599	Coronavirus infectious disease	1.35E−11	42.894222
	DOID:9001645	Coronaviridae Infections	1.37E−11	42.86042

Table 6 presents SCADDx and LOADDx results at $n = 1$. It is of course more challenging to correctly detect the single most likely disease ($n = 1$) than for it to be in a list of 10 most likely candidates ($n = 10$). However, SCADDx can still achieve an average accuracy of 88.99% using the CTD KB and 91.38% using the DisGeNet KB within 72 h of infection. Table 6 presents the t-test results, which indicate that SCADDx with both KBs and LOADDx with the DisGeNet KB outperformed *k*-NN significantly. Overall, these findings suggest that SCADDx and LOADDx are reliable tools for detecting infection, even in challenging circumstances.

Table 7 presents the mean accuracy of various algorithms obtained through LOOCV. The results indicate that SCADDx and LOADDx with the DisGeNet KB consistently outperformed the existing machine learning algorithms on all the datasets. To determine the overall performance of the algorithms, we computed the average accuracy across all the datasets (see Table 7). According to the paired t-test results, SCADDx and LOADDx using both KBs achieved significantly higher accuracy than all the existing algorithms, with a *p* value < 0.01 (see Table 7).

Based on the results shown in Tables 5, 6 and 7, it can be concluded that overall SCADDx with both the KBs performed very well on all the datasets. This shows that the use of magnitudes of MDEGs/LDEGs in combination with KB can help in gaining better results. In case of Dataset 3 and Dataset 4, LOADDx also performed similar to SCADDx but it did not perform so well on other datasets. This is due to the fact that LOADDx doesn’t utilize magnitudes of MDEGs/LDEGs. The reason why the machine learning models couldn’t perform so well is that they do not exploit KB for prediction. They only use the gene expression data. This suggests that the use of KB can help in better disease prediction.

We conducted Gene Set Enrichment Analysis (GSEA) [72] by selecting the most important 16 genes (listed in Table 8) across all four gene expression datasets used in this study. We identified these genes by taking the intersection of the top MDEGs (P) across all four datasets used in disease prediction with SCADDx. To perform GSEA, we used the Multi-Ontology Enrichment Tool (MOET),^10^ a web-based enrichment analysis tool that supports multiple ontologies for multiple species, including humans. The results of GSEA, presented in Table 8, show that the 16 genes are strongly associated with Human Influenza. Moreover, the top 10 terms produced by GSEA that are associated with these 16 genes are closely related to RVI. These findings suggest that these 16 genes can serve as important biomarkers and play a crucial role in precision medicine.

## Conclusions and future work

In this paper, we have proposed two new algorithms, LOADDx and SCADDx, to combine patients’ gene expression data with a KB. LOADDx and SCADDx can produce a short personalised ranked list of the most likely diseases with other entities linked with them in the KB for each patient at a requested time-point. We have discovered how a patient’s Least Differentially Expressed Genes (LDEGs) along with Most Differentially Expressed Genes (MDEGs) can help in disease diagnosis in the presence of a KB. We identified the potential of LDEGs in such settings and used them for disease diagnosis in combination with KB. We showed how KBs that do not include link strength information can be used to infer the strength of links in a patient-specific manner, using the patient’s gene expression profile. We evaluated both SCADDx and LOADDx using two KBs and four real-world gene expression datasets of respiratory viral infections caused by 19 subtypes of Influenza-like viruses. Additionally, we compared the performance of these algorithms with five existing machine learning algorithms. Our results showed that both SCADDx and LOADDx consistently outperformed the existing machine learning algorithms, as demonstrated by both validation approaches, namely LOOCV and single internal validation set approach.

SCADDx and LOADDx can predict the diseases that a person is most likely to have, at an early stage, with high accuracy, by combining their gene expression data with a KB. We have also provided the visualisation of results that can show the MDEGs and LDEGs associated with the disease in KB for each subject. Moreover, for each patient, the proposed algorithms can show the changes in gene expression values of the most affected genes together with the computed disease scores and can produce a ranked personalized list of the most likely diseases along with other entities linked with them in the KB, which can support health care professionals in their decision-making.

In future, we intend to perform experiments on subjects who are suffering from multiple diseases. We will also explore how the incorporation of more contextual links in KB can improve the accuracy of disease diagnosis.

### Supplementary Information

**Additional file 1.** Additional Figures and Tables.

## Data Availability

All the implementation of the algorithms and statistical analysis are done using R programming language. Supplementary data and source codes are available online at https://github.com/GhanshyamVerma/Disease-Diagnosis-Assistants.

## Footnotes

1 https://www.disgenet.org/.

2 https://xgboost.readthedocs.io/en/stable/parameter.html.

3 https://www.ncbi.nlm.nih.gov/geo/query/acc.cgi?acc=GSE73072.

4 https://www.ncbi.nlm.nih.gov/geo/query/acc.cgi?acc=GSE68310.

5 https://www.ncbi.nlm.nih.gov/geo/query/acc.cgi?acc=GSE90732.

6 https://www.ncbi.nlm.nih.gov/geo/query/acc.cgi?acc=GSE61754.

7 https://www.disgenet.org/.

8 https://www.disgenet.org/rdf.

9 https://ctdbase.org/.

10 https://rgd.mcw.edu/rgdweb/enrichment/start.html.
