# Supplementary material for: Enabling personalised disease diagnosis by combining a patient’s time-specific gene expression profile with a biomedical knowledge base
Source: BMC Bioinformatics. 2024 Feb 7;25:62. doi: 10.1186/s12859-024-05674-0 (PMC10848462; doi:10.1186/s12859-024-05674-0)
Supplement: Supplementary file 1 — Additional file 1. Additional Figures and Tables. [file 12859_2024_5674_MOESM1_ESM.pdf]

Supplementary Material for "Enabling personalised disease diagnosis by combining a patient's time-specific gene expression profile with a biomedical knowledge base"

Ghanshyam Verma, Dietrich Rebholz-Schuhmann and Michael G. Madden

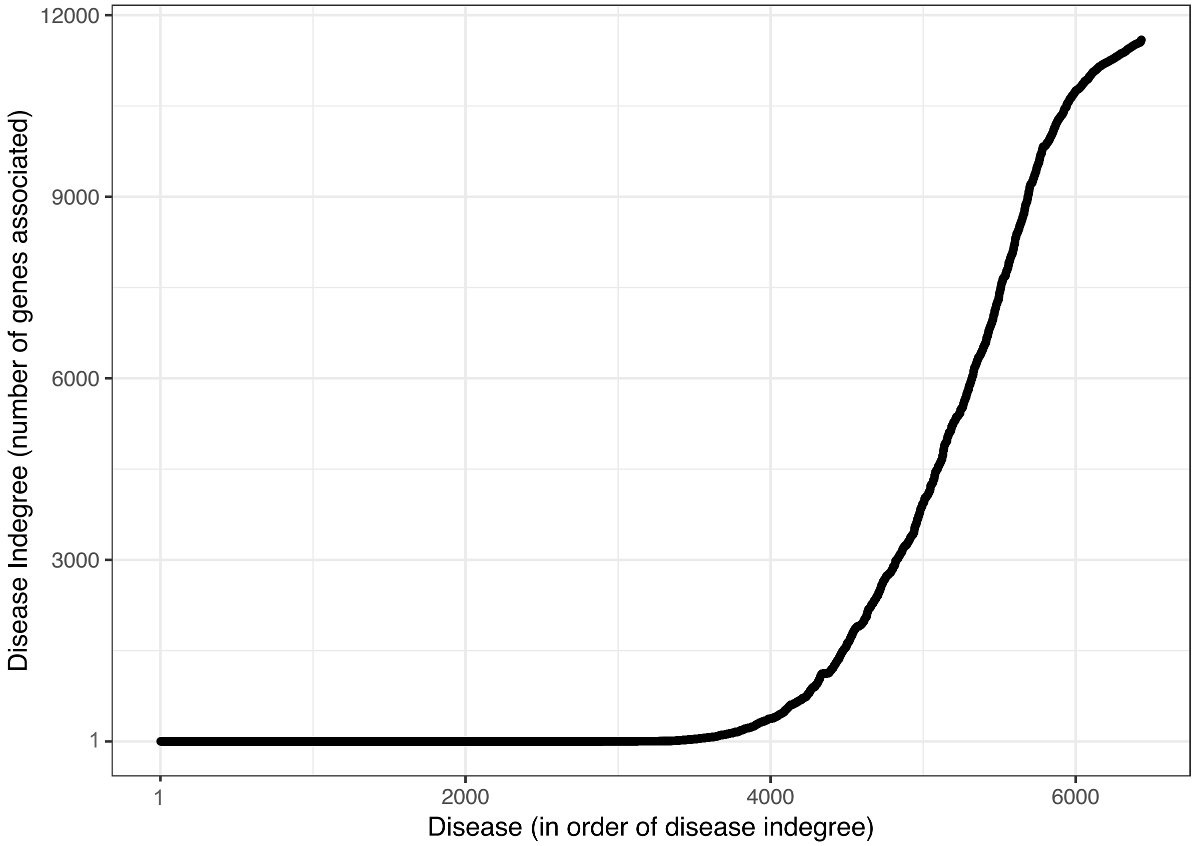

Fig S1. Plot of CTD KB disease indegree. X-axis shows all the diseases in ascending order of their indegree. Y-axis shows the number of genes associated with a particular disease (disease indegree).

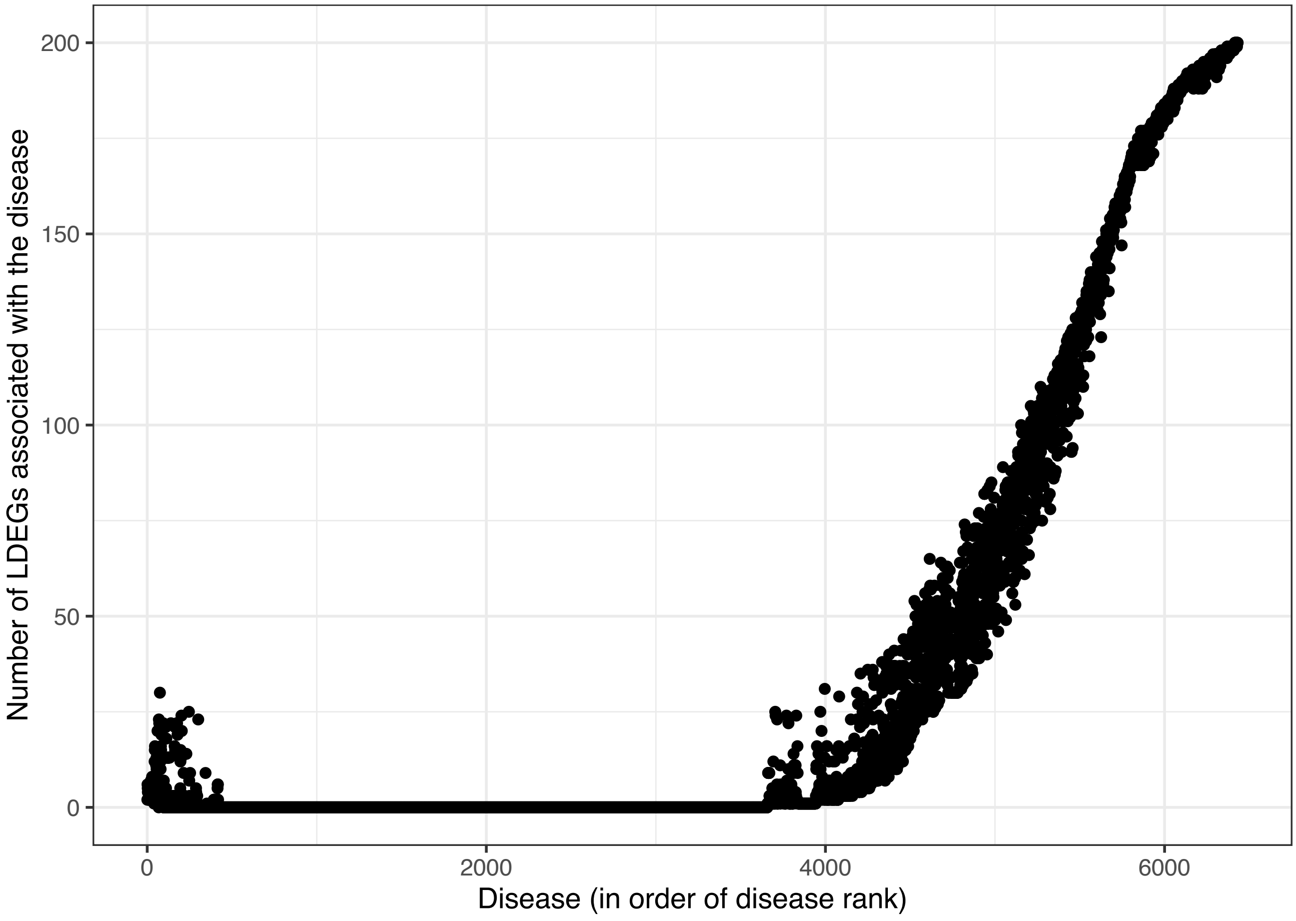

Fig S2. Plot of number of LDEGs associated with diseases in CTD KB. The LDEGs belong to subject 1 of Dataset 2. X-axis shows all CTD KB diseases in order of their rank assigned by SCADDx to subject 1 of Dataset 2. Y-axis shows the subject's number of LDEGs associated with the corresponding ranked disease.

Table S1. Sample of results for the first 5 subjects of Testset 1a (GSE73072) using SCADDx on CTD KB. Showing top 5 diseases for each subject with most affected 5 genes. Parameter values: $P = 100$, $Q = 175$, $m = 5$, time $t_D \simeq 60$ hours.

Subject ID	Top 5 Genes (abs(ΔG))	Disease Name	Disease Score	Disease Probability (Softmax)	Predicted Class Label	True Class Label
1	PDIA3 (0.22)<br>RALGDS (0.21)<br>TNKS2 (0.21)<br>NCKAP1L (0.21)<br>ANXA6 (0.20)	Dysentery, Bacillary	0.73	21.81%	Not RVI	Not RVI
		Colonic Diseases, Functional	0.64	19.99%		
		Esophageal Motility Disorders	0.64	19.99%		
		Hypochondriasis	0.61	19.35%		
		Encephalitis, Herpes Simplex	0.58	18.86%		
2	APBB1IP (0.33)<br>HBB (0.29)<br>TAGLN2 (0.27)<br>USP34 (0.216)<br>FAM106A (0.25)	Subdural Effusion	0.65	23.33%	Not RVI	RVI
		Dysentery, Bacillary	0.65	23.33%		
		Penile Neoplasms	0.43	18.77%		
		Hepatitis, Viral, Animal	0.35	17.36%		
		Antley-Bixler Syndrome Phenotype	0.34	17.21%		
3	IFI27 (0.44)<br>IFI44L (0.30)<br>SPATS2L (0.29)<br>IFI44 (0.29)<br>RSAD2 (0.29)	Respiratory Viral Infection	9.65	92.66%	RVI	RVI
		Failure to Thrive	5.82	2.02%		
		Paraparesis, Tropical Spastic	5.69	1.77%		
		Mitochondrial myopathy with lactic acidosis	5.69	1.77%		
		Retroviridae Infections	5.69	1.77%		
4	CSTA (0.16)<br>KLRB1 (0.13)<br>NDUFA1 (0.12)<br>ATP5F1 (0.12)<br>RPL36AP37 (0.12)	Extensively Drug-Resistant Tuberculosis	0.39	22.7%	Not RVI	Not RVI
		Phantom Limb	0.23	19.4%		
		Trochlear Nerve Diseases	0.23	19.4%		
		Alexander Disease	0.22	19.3%		
		Epilepsy, Benign Neonatal	0.22	19.3%		
5	DMXL1 (0.22)<br>BMI1 (0.20)<br>MYBL1 (0.19)<br>ZBTB11 (0.17)<br>PLEKHF2 (0.17)	Osteosclerosis	0.54	20.85%	Not RVI	Not RVI
		Echolalia	0.53	20.78%		
		Contracture	0.50	20.03%		
		Esophageal Stenosis	0.49	19.81%		
		Appendiceal Neoplasms	0.42	18.52%		

Table S2. Sample of results for the first 5 subjects of Testset 1b (GSE73072) using SCADDx on CTD KB. Showing top 5 diseases for each subject with most affected 5 genes. Parameter values: $P = 100$, $Q = 175$, $m = 5$, time $t_D \simeq 60$ hours.

Subject ID	Top 5 Genes (abs(ΔG))	Disease Name	Disease Score	Disease Probability (Softmax)	Predicted Class Label	True Class Label
1	RSAD2 (0.54)<br>IFIT1 (0.53)<br>IFI44L (0.52)<br>LAMP3 (0.49)<br>IFI44 (0.44)	Respiratory Viral Infection	16.81	99.98%	RVI	RVI
		Panuveitis	6.94	0.01%		
		Fasciitis, Plantar	6.94	0.01%		
		Neuromyelitis Optica	6.94	0.01%		
		Orbital Pseudotumor	6.94	0.01%		
2	SPON2 (0.16)<br>KIR2DL3 (0.14)<br>DDIT4 (0.13)	Subdural Effusion	0.62	25.19%	Not RVI	Not RVI
		Bacteriuria	0.35	19.17%		

	ANXA3 (0.12)<br>CLIC3 (0.12)	Dysentery, Bacillary	0.35	19.17%		
		Hymenolepiasis	0.30	18.23%		
		Cat Diseases	0.30	18.23%		
3	LAMP3 (0.54)<br>RSAD2 (0.49)<br>IFIT1 (0.48)<br>ISG15 (0.44)<br>SERPING1 (0.43)	Respiratory Viral Infection	9.65	17.30%	RVI	RVI
		Paraparesis, Tropical Spastic	5.82	9.63%		
		Mitochondrial encephalopathy	5.69	9.40%		
		Mitochondrial myopathy with lactic acidosis	5.69	9.40%		
		Retroviridae Infections	5.69	9.40%		
4	RSAD2 (0.37)<br>IFIT1 (0.32)<br>IFI44L (0.29)<br>CCL8 (0.27)<br>IFI44 (0.27)	Respiratory Viral Infection	8.88	98.59%	RVI	RVI
		Severe Acute Respiratory Syndrome	3.43	0.42%		
		Panuveitis	3.17	0.33%		
		Fasciitis, Plantar	3.17	0.33%		
		Neuromyelitis Optica	3.17	0.33%		
5	MYBL1 (0.17)<br>C1D (0.15)<br>ZFYVE16 (0.15)<br>CASD1 (0.14)<br>CHORDC1 (0.13)	Hyperglycinemia, Nonketotic	0.44	20.52%	Not RVI	RVI
		Ochronosis	0.43	20.31%		
		Anemia, Refractory	0.41	19.72%		
		Chromosome 17 deletion	0.41	19.72%		
		Chromosome 5q Deletion Syndrome	0.41	19.72%		

Table S3. Sample of results for the first 5 subjects of Testset 2a (GSE68310) using SCADDc on CTD KB. Showing top 5 diseases for each subject with most affected 5 genes. Parameter values: $P = 150$, $Q = 300$, $m = 5$, time t_D = day 2.

Subject ID	Top 5 Genes (abs(ΔG))	Disease Name	Disease Score	Disease Probability (Softmax)	Predicted Class Label	True Class Label
1	IFI27 (0.65),<br>IFI44L (0.63),<br>ISG15 (0.61),<br>RSAD2 (0.61),<br>IFI44 (0.53)	Respiratory Viral Infection	21.58	99.99%	RVI	RVI
		Severe Acute Respiratory Syndrome	6.23	0%		
		Alopecia universalis	5.92	0%		
		Panuveitis	5.89	0%		
		Orbital Pseudotumor	5.89	0%		
2	RSAD2 (0.62),<br>ISG15 (0.62),<br>IFITM3 (0.61),<br>IFI27 (0.58),<br>IFI44L (0.58)	Respiratory Viral Infection	19.89	99.99%	RVI	RVI
		Rupture, Spontaneous	6.23	0%		
		Panuveitis	6.16	0%		
		Orbital Pseudotumor	6.16	0%		
		Fibrous Dysplasia of Bone	6.09	0%		
3	RSAD2 (0.70),<br>ISG15 (0.67),<br>IFITM3 (0.66),<br>IFI44L (0.65),<br>HERC5 (0.63)	Respiratory Viral Infection	22.76	99.99%	RVI	RVI
		Panuveitis	9.15	0%		
		Neuromyelitis Optica	9.15	0%		
		Orbital Pseudotumor	9.15	0%		
		Fasciitis, Plantar	8.45	0%		
4	ISG15 (0.61),<br>RSAD2 (0.57),<br>CXCL10 (0.55),<br>IFI44L (0.54),<br>IFITM3 (0.53)	Respiratory Viral Infection	18.92	99.99 %	RVI	RVI
		Pulmonary Aspergillosis	8.29	0%		
		Panuveitis	8.86	0%		
		Fasciitis, Plantar	8.18	0%		
		Neuromyelitis Optica	8.18	0%		
5	IFITM3 (0.60),<br>ISG15 (0.52),<br>IFI44L (0.52),<br>RSAD2 (0.52),<br>HERC5 (0.47)	Respiratory Viral Infection	12.73	98.08%	RVI	RVI
		Panuveitis	7.55	0.55%		
		Orbital Pseudotumor	7.55	0.55%		
		Severe Acute Respiratory Syndrome	7.55	0.51%		
		PanNeuromyelitis Optica	6.95	0.30%		

Table S4. Sample of results for the first 5 subjects of Testset 2b (GSE68310) using SCADDc on CTD KB. Showing top 5 diseases for each subject with most affected 5 genes. Parameter values: $P = 150$, $Q = 300$, $m = 5$, time t_D = day 2.

Subject ID	Top 5 Genes (abs(ΔG))	Disease Name	Disease Score	Disease Probability (Softmax)	Predicted Class Label	True Class Label
1	IFI27 (0.75), IFI44L (0.52), LY6E (0.41), IFITM3 (0.37), ALPL (0.36)	Respiratory Viral Infection	8.06	69.64%	RVI	RVI
		Severe Acute Respiratory Syndrome	6.05	9.28%		
		Acquired angioedema	5.90	7.96%		
		Pulmonary Aspergillosis	5.76	6.93%		
		Panuveitis	5.64	6.16%		
2	RSAD2 (0.63), ISG15 (0.62), IFITM3 (0.62), IFI44L (0.61), HERC5 (0.57)	Respiratory Viral Infection	18.48	99.99%	RVI	RVI
		Panuveitis	7.50	0%		
		Orbital Pseudotumor	7.50	0%		
		Pulmonary Aspergillosis	7.45	0%		
		Toxocariasis	7.07	0%		
3	ISG15 (0.81), HERC5 (0.69), MX1 (0.69), RSAD2 (0.69), IFITM3 (0.67)	Respiratory Viral Infection	25.98	100%	RVI	RVI
		Panuveitis	7.31	0%		
		Neuromyelitis Optica	7.31	0%		
		Collagen Diseases	7.22	0%		
		Lymphohistiocytosis, Hemophagocytic	7.22	0%		
4	IFITM3 (0.78), ISG15 (0.68), RSAD2 (0.64), IFI27 (0.63), IFI44L (0.59)	Respiratory Viral Infection	18.42	99.98%	RVI	RVI
		Panuveitis	8.27	0%		
		Fasciitis, Plantar	8.27	0%		
		Neuromyelitis Optica	8.27	0%		
		Orbital Pseudotumor	8.27	0%		
5	IFI27 (0.71), LY6E (0.53), ISG15 (0.53), IFI44L (0.52), IFITM3 (0.51)	Respiratory Viral Infection	13.96	99.97%	RVI	RVI
		Paraparesis, Tropical Spastic	4.15	0%		
		AIDS-Related Complex	4.15	0%		
		Mitochondrial myopathy with lactic acidosis	4.15	0%		
		Retroviridae Infections	4.15	0%		

Table S5. Sample of results for all the subjects of Testset 3 (GSE90732) using SCADDx on CTD KB. Showing top 5 diseases for each subject with most affected 5 genes. Parameter values: $P = 25$, $Q = 25$, $m = 5$, time t_D= 72 hours.

Subject ID	Top 5 Genes (abs(ΔG))	Disease Name	Disease Score	Disease Probability (Softmax)	Predicted Class Label	True Class Label
1	CCL2 (0.59), CCL8 (0.59), RSAD2 (0.58), OTOF (0.55), CXCL10 (0.54)	Respiratory Viral Infection	8.94	26.13%	RVI	RVI
		Spinal Disease	8.83	23.33%		
		Carcinoma, Adenosquamous	8.70	20.53%		
		Osteonecrosis	8.43	15.69%		
		Polymyositis	8.43	14.29%		
2	RSAD2 (0.59), CXCL10 (0.56), IFI44 (0.53), IFIT3 (0.52), DDX60 (0.48)	Respiratory Viral Infection	8.43	72.33%	RVI	RVI
		Pulpitis	6.31	8.69%		
		Ciliophora Infections	6	6.32%		
		Formaldehyde poisoning	6	6.32%		
		Warts	6	6.32%		
3	CXCL10 (0.61), IFI27 (0.56),	Respiratory Viral Infection	7.46	60.60%	RVI	RVI
		Macular Edema	5.84	11.99%		

	CCL8 (0.48), OTOF (0.48), SERPING1 (0.47)	Encephalomyelitis, Acute Disseminated	5.70	10.37%		
		Sjogren's Syndrome	5.59	9.30%		
		Flatulence	5.40	7.72%		
4	RSAD2 (0.58), CXCL10 (0.54), CCL8 (0.51), ISG15 (0.49), SERPING1 (0.48)	Respiratory Viral Infection	9.10	45.97%	RVI	RVI
		Hearing Loss, Sudden	8.35	21.68%		
		Encephalomyelitis, Acute Disseminated	7.70	11.34%		
		Anti-Glomerular Basement Membrane Disease	7.70	11.28%		
		Osteonecrosis	7.55	9.7%		

Table S6. Sample of results for the first 5 subjects of Testset 4 (GSE61754) using SCADDx on CTD KB. Showing top 5 diseases for each subject with most affected 5 genes. Parameter values: $P = 25$, $Q = 25$, $m = 5$, time t_D= 48 hours.

Subject ID	Top 5 Genes (abs(ΔG))	Disease Name	Disease Score	Disease Probability (Softmax)	Predicted Class Label	True Class Label
1	ARF4 (0.22), ATG4B (0.21), PSMC4 (0.21), ARHGAP26 (0.20), CNBP (0.19)	Tumor Lysis Syndrome	1.73	37.06%	Not RVI	Not RVI
		Language Disorders	0.89	16.09%		
		Tobacco Use Disorder	0.88	15.91%		
		Sweet Syndrome	0.86	15.54%		
		Fibroma	0.85	15.38%		
2	OTUD3 (0.13), GALR3 (0.13), SERPINA10 (0.13), B3GAT3 (0.13), TJP1 (0.12)	Enterocolitis, Necrotizing	1.07	22.18%	Not RVI	Not RVI
		Hypopigmentation	1.02	21.11%		
		Fat Necrosis	0.93	19.28%		
		Cadmium Poisoning	0.90	18.82%		
		Periodontal Diseases	0.89	18.58%		
3	PPIG (0.20), PDK4 (0.19), ARID4A (0.19), LARP7 (0.18), PSMC6 (0.18)	Neoplasms, Radiation-Induced	1.90	25.57%	Not RVI	Not RVI
		Ataxia Telangiectasia	1.63	19.52%		
		Myotonia	1.61	19.21%		
		Common Cold	1.58	18.62%		
		Blepharoptosis	1.49	17.05%		
4	IFIT1 (0.34), RSAD2 (0.32), IFI44L (0.32), HERC5 (0.28), ISG15 (0.27)	Respiratory Viral Infection	5.13	56.27%	RVI	RVI
		Flatulence	3.74	13.98%		
		Virilism	3.51	11.06%		
		Actinomycetales Infections	3.34	9.33%		
		Giant Cell Arteritis	3.34	9.33%		
5	RSAD2 (0.53), LAMP3 (0.47), IFI44L (0.47), OAS3 (0.43), HERC5 (0.40)	Respiratory Viral Infection	9.05	85.49%	RVI	RVI
		Acquired Hyperostosis Syndrome	5.97	3.95%		
		Lipomatosis	5.93	3.77%		
		Carcinoma, Adenosquamous	5.83	3.41%		
		Pregnancy Complications, Infectious	5.81	3.35%		

Table S7. Sample of results for the first 5 subjects of Testset 1a (GSE73072) using LOADDx on CTD KB. Showing top 5 diseases for each subject with most affected 5 genes. Parameter values: $P = 25$, $Q = 225$, $m = 5$, time $t_D \simeq 60$ hours.

Subject ID	Top 5 Genes (abs(ΔG))	Disease Name	Disease Score	Disease Probability (Softmax)	Predicted Class Label	True Class Label
1	PDIA3 (0.22), RALGDS (0.21), TNKS2 (0.21), NCKAP1L (0.21), ANXA6 (0.20)	Hypochondriasis	3.67	24.06%	Not RVI	Not RVI
		Dysentery, Bacillary	3.67	24.06%		
		Hypersplenism	3.34	17.29%		
		Latent Tuberculosis	3.34	17.29%		
		Steatorrhea	3.34	17.29%		
2	APBB1IP (0.33), HBB (0.29), TAGLN2 (0.27), USP34 (0.26), FAM106A (0.25)	Dysentery, Bacillary	3.34	28.08%	Not RVI	RVI
		Antley-Bixler Syndrome Phenotype	2.89	17.97%		
		Candidiasis, Cutaneous	2.89	17.97%		
		Puerperal Infection	2.89	17.97%		
		Myopathies, Structural, Congenital	2.89	17.97%		
3	IFI27 (0.49), IFI44L (0.44), SPATS2L (0.43), IFI44 (0.42), RSAD2 (0.39)	Respiratory Viral Infection	6.20	54.22%	RVI	RVI
		Panuveitis	4.64	11.44%		
		Fasciitis, Plantar	4.64	11.44%		
		Fibrous Dysplasia of Bone	4.64	11.44%		
		Neuromyelitis Optica	4.64	11.44%		
4	CSTA (0.16), KLRB1 (0.13), NDUFA1 (0.12), ATP5F1 (0.12), RPL36AP37 (0.12)	Dysentery, Bacillary	2.89	20%	Not RVI	Not RVI
		Alopecia universalis	2.89	20%		
		Carcinoma, Intraductal, Noninfiltrating	2.89	20%		
		Exfoliative Ichthyosis, Autosomal Recessive, Ichthyosis Bullosa of Siemens-like	2.89	20%		
		Mitochondrial Complex III Deficiency	2.89	20%		
5	DMXL1 (0.22), BMI1 (0.20), MYBL1 (0.19), ZBTB11 (0.17), PLEKHF2 (0.17)	Carcinoma, Adenoid Cystic	2.97	21.29%	Not RVI	Not RVI
		Appendiceal Neoplasms	2.89	19.67%		
		Foot Injuries	2.89	19.67%		
		Hand Injuries	2.89	19.67%		
		Disorder of Sex Development, 46,XY	2.89	19.67%		

Table S8. Sample of results for the first 5 subjects of Testset 1b (GSE73072) using LOADDx on CTD KB. Showing top 5 diseases for each subject with most affected 5 genes. Parameter values: $P = 25$, $Q = 225$, $m = 5$, time $t_D \simeq 60$ hours.

Subject ID	Top 5 Genes (abs(ΔG))	Disease Name	Disease Score	Disease Probability (Softmax)	Predicted Class Label	True Class Label
1	RSAD2 (0.54), IFIT1 (0.53), IFI44L (0.52), LAMP3 (0.48), IFI44 (0.43)	Respiratory Viral Infection	6.06	54.39%	RVI	RVI
		Collagen Diseases	4.49	11.40%		
		Epileptic encephalopathy, Lennox-Gastaut type	4.49	11.40%		
		Lymphohistiocytosis, Hemophagocytic	4.49	11.40%		
		Mitochondrial cytopathy	4.49	11.40%		
2	SPON2 (0.33), KIR2DL3 (0.29), DDIT4 (0.27), ANXA3 (0.26), CLIC3 (0.25)	Hymenolepiasis	3.34	20 %	Not RVI	Not RVI
		Cat Diseases	3.34	20 %		
		Oxyurida Infections	3.34	20 %		
		Tick Infestations	3.34	20 %		
		Trichuriasis	3.34	20 %		
3	LAMP3 (0.53), RSAD2 (0.49), IFIT1 (0.48),	Respiratory Viral Infection	6.39	63.97 %	RVI	RVI
		Nephrogenic Fibrosing Dermopathy	4.55	10.19 %		
		Paraparesis, Tropical Spastic	4.38	8.61 %		

	ISG15 (0.44), SERPING1 (0.42)	Mitochondrial encephalopathy	4.38	8.61 %		
		Mitochondrial myopathy with lactic acidosis	4.38	8.61 %		
4	RSAD2 (0.37), IFIT1 (0.31), IFI44L (0.29), CCL8 (0.26), IFI44 (0.26)	Respiratory Viral Infection	6.39	61.44%	RVI	RVI
		Foot-and-Mouth Disease	4.72	4.72%		
		Vesicular Stomatitis	4.72	4.72%		
		Nephrogenic Fibrosing Dermopathy	4.37	4.37%		
		Panuveitis	4.24	4.24%		
5	MYBL1 (0.22), C1D (0.20), ZFYVE16 (0.19), CASD1 (0.17), CHORDC1 (0.17)	Mitochondrial Encephalomyopathies	2.89	20%	Not RVI	RVI
		Medium chain acyl CoA dehydrogenase deficiency	2.89	20%		
		Mental Retardation, X-Linked 63	2.89	20%		
		Glycogen Storage Disease Type II	2.89	20%		
		Glycogen Storage Disease Type III	2.89	20%		

Table S9. Sample of results for the first 5 subjects of Testset 2a (GSE68310) using LOADDx on CTD KB. Showing top 5 diseases for each subject with most affected 5 genes. Parameter values: $P = 50$, $Q = 300$ genes, $m = 5$, time t_D= day 2.

**Subject ID**	**Top 5 Genes (abs(ΔG))**	**Disease Name**	**Disease Score**	**Disease Probability (Softmax)**	**Predicted Class Label**	**True Class Label**
1	IFI27 (0.65), IFI44L (0.63), ISG15 (0.61), RSAD2 (0.61), IFI44 (0.53)	Respiratory Viral Infection	6.70	85.26%	RVI	RVI
		Alopecia universalis	3.91	5.21%		
		Foot-and-Mouth Disease	3.44	3.27%		
		Vesicular Stomatitis	3.44	3.27%		
		Severe Acute Respiratory Syndrome	3.34	2.96%		
2	RSAD2 (0.63), ISG15 (0.62), IFITM3 (0.61), IFI27 (0.58), IFI44L (0.58)	Respiratory Viral Infection	5.80	65.53%	RVI	RVI
		Alopecia universalis	4.10	11.99%		
		Foot-and-Mouth Disease	3.69	7.93%		
		Vesicular Stomatitis	3.69	7.93%		
		Panuveitis	3.50	6.59%		
3	RSAD2 (0.70), ISG15 (0.67), IFITM3 (0.66), IFI44L (0.65), HERC5 (0.63),	Respiratory Viral Infection	6.21	68.17%	RVI	RVI
		Panuveitis	4.13	8.49%		
		Neuromyelitis Optica	4.13	8.49%		
		Orbital Pseudotumor	4.13	8.49%		
		Fasciitis, Plantar	3.84	6.34%		
4	ISG15 (0.61), RSAD2 (0.57), CXCL10 (0.55), IFI44L (0.54), IFITM3 (0.53)	Respiratory Viral Infection	6.57	63.32%	RVI	RVI
		Alopecia universalis	4.89	11.82%		
		Panuveitis	4.54	8.28%		
		Fasciitis, Plantar	4.54	8.28%		
		Neuromyelitis Optica	4.54	8.28%		
5	RSAD2 (0.60), ISG15 (0.52), IFI44L (0.52), RSAD2 (0.51), HERC5 (0.47)	Panuveitis	5.23	26.66%	RVI	RVI
		Orbital Pseudotumor	5.23	26.66%		
		Respiratory Viral Infection	4.89	18.97%		
		Severe Acute Respiratory Syndrome	4.62	14.40%		
		Neuromyelitis Optica	4.54	13.28%		

Table S10. Sample of results for the first 5 subjects of Testset 2b (GSE68310) using LOADDx on CTD KB. Showing top 5 diseases for each subject with most affected 5 genes. Parameter values: $P = 50, Q = 300$ genes, $m = 5$, time t_D= day 2.

Subject ID	Top 5 Genes (abs(ΔG))	Disease Name	Disease Score	Disease Probability (Softmax)	Predicted Class Label	True Class Label
1	IFI27 (0.75), IFI44L (0.52), LY6E (0.41), IFITM3 (0.37), ALPL (0.36)	Severe Acute Respiratory Syndrome	5.07	21.41%	Not RVI	RVI
		Panuveitis	4.98	19.64%		
		Acquired angioedema	4.98	19.64%		
		Orbital Pseudotumor	4.98	19.64%		
		Pulmonary Aspergillosis	4.98	19.64%		
2	RSAD2 (0.63), ISG15 (0.62), IFITM3 (0.62), IFI44L (0.61), HERC5 (0.57)	Respiratory Viral Infection	5.91	54.55%	RVI	RVI
		Alopecia universalis	4.80	17.99%		
		Foot-and-Mouth Disease	4.27	10.56%		
		Vesicular Stomatitis	4.27	10.56%		
		Panuveitis	3.75	6.32%		
3	ISG15 (0.81), HERC5 (0.69), MX1 (0.69), RSAD2 (0.69), IFITM3 (0.67),	Respiratory Viral Infection	5.74	71.32%	RVI	RVI
		Collagen Diseases	3.44	7.16%		
		Lymphohistiocytosis, Hemophagocytic	3.44	7.16%		
		Mitochondrial cytopathy	3.44	7.16%		
		Pars Planitis	3.44	7.16%		
4	IFITM3 (0.78), ISG15 (0.68), RSAD2 (0.64), IFI27 (0.63), IFI44L (0.59)	Respiratory Viral Infection	5.40	51.21%	RVI	RVI
		Autoimmune Lymphoproliferative Syndrome	4.10	13.93%		
		Alopecia universalis	4.01	12.66%		
		Panuveitis	3.87	11.09%		
		Fasciitis, Plantar	3.87	11.09%		
5	IFI27 (0.71), LY6E (0.53), ISG15 (0.53), IFI44L (0.52), IFITM3 (0.51)	Respiratory Viral Infection	5.64	61.61%	RVI	RVI
		Dysentery, Amebic	4.27	15.58%		
		Alopecia universalis	3.79	9.63%		
		Absence of Tibia	3.46	6.96%		
		Paraparesis, Tropical Spastic	3.35	6.20%		

Table S11. Sample of results for all the subjects of Testset 3 (GSE90732) using LOADDx on CTD KB. Showing top 5 diseases for each subject with most affected 5 genes. Parameter values: $P = 25, Q = 25$, $m = 5$, time t_D= 72 hours.

Subject ID	Top 5 Genes (abs(ΔG))	Disease Name	Disease Score	Disease Probability (Softmax)	Predicted Class Label	True Class Label
1	CCL2 (0.59), CCL8 (0.59), RSAD2 (0.58), OTOF (0.55), CXCL10 (0.54)	Carcinoma, Adenosquamous	4.30	23.91%	RVI	RVI
		Respiratory Viral Infection	4.30	23.91%		
		Spinal Diseases	4.00	17.70%		
		Polymyositis	4.00	17.70%		
		Pregnancy Complications, Infectious	3.95	16.74%		
2	RSAD2 (0.59), CXCL10 (0.56), IFI44 (0.53), IFIT3 (0.52), DDX60 (0.48)	Respiratory Viral Infection	4.30	44.21%	RVI	RVI
		Radiation Injuries, Experimental	3.33	16.66%		
		Proctocolitis	3.18	14.36%		
		Hemolytic-Uremic Syndrome	3.03	12.37%		
		Lupus Erythematosus, Discoid	3.03	12.37%		
3	CXCL10 (0.61), IFI27 (0.56), CCL8 (0.48), OTOF (0.48), SERPING1 (0.47)	Respiratory Viral Infection	4.30	29.58%	RVI	RVI
		Candidiasis, Vulvovaginal	3.78	17.60%		
		Urinary Bladder Calculi	3.78	17.60%		
		Activated Protein C Resistance	3.78	17.60%		
		Biliary Dyskinesia	3.78	17.60%		

4	RSAD2 (0.58), CXCL10 (0.54), CCL8 (0.51), ISG15 (0.49), SERPING1 (0.48)	Respiratory Viral Infection	5.00	35.57%	RVI	RVI
		Hearing Loss, Sudden	4.51	21.65%		
		Helicobacter Infections	4.30	17.67%		
		Encephalomyelitis, Acute Disseminated	4.12	14.69%		
		Anti-Glomerular Basement Membrane Disease	3.77	10.40%		

Table S12. Sample of results for the first 5 subjects of Testset 4 (GSE61754) using LOADDx on CTD KB. Showing top 5 diseases for each subject with most affected 5 genes. Parameter values: $P = 25$, $Q = 50$, $m = 5$, time t_D= 48 hours.

Subject ID	Top 5 Genes (abs(ΔG))	Disease Name	Disease Score	Disease Probability (Softmax)	Predicted Class Label	True Class Label
1	ARF4 (0.22), ATG4B (0.21), PSMC4 (0.21), ARHGAP26 (0.20), CNBP (0.19)	Leg Ulcer	2.18	21.19%	Not RVI	Not RVI
		Sweet Syndrome	2.18	21.19%		
		Hip Fractures	2.18	21.19%		
		Paroxysmal nonkinesigenic	2.18	21.19%		
		Histiocytic Sarcoma	1.85	15.23%		
2	OTUD3 (0.13), GALR3 (0.13), SERPINA10 (0.13), B3GAT3 (0.13), TJP1 (0.12)	Polymyalgia Rheumatica	1.85	22.96%	Not RVI	Not RVI
		Chromosome 22, microdeletion 22 q11	1.85	22.96%		
		Pulmonary Valve Stenosis	1.73	20.46%		
		AIDS-Related Complex	1.54	16.80%		
		Burning Mouth Syndrome	1.54	16.80%		
3	PPIG (0.20), PDK4 (0.19), ARID4A (0.19), LARP7 (0.18), PSMC6 (0.18)	Histiocytic Sarcoma	2.18	23.69%	Not RVI	Not RVI
		Diabetic Neuropathies	2.15	23.15%		
		Hand Deformities, Congenital	1.96	19.08%		
		Castleman Disease	1.85	17.03%		
		Choanal Atresia	1.85	17.03%		
4	IFIT1 (0.34), RSAD2 (0.32), IFI44L (0.32), HERC5 (0.28), ISG15 (0.27)	Respiratory Viral Infection	5.68	57.17%	RVI	RVI
		Actinomycetales Infections	4.00	10.70%		
		Giant Cell Arteritis	4.00	10.70%		
		Neoplasms, Second Primary	5.68	10.70%		
		Dementia, Vascular	4.00	10.70%		
5	RSAD2 (0.53), LAMP3 (0.47), IFI44L (0.47), OAS3 (0.43), HERC5 (0.40)	Respiratory Viral Infection	6.45	78.92%	RVI	RVI
		Lipomatosis	3.91	6.19%		
		Severe Acute Respiratory Syndrome	3.74	5.26%		
		Carcinoma, Adenosquamous	3.72	5.12%		
		Panuveitis	3.59	4.50%		

Table S13. List of all the viruses which caused respiratory viral infection with number of subjects infected for all the four gene expression data sets used in this study.

Serial	Name of Virus / Virus Subtypes	Number of Infected Subjects	Virus Group (based on number of subjects infected with a virus)	Dataset
1.	H1N1 virus	30	H1N1	Dataset 1 (15)<br>Dataset 3 (15)
2.	H3N2 virus	30	H3N2	Dataset 1 (17)<br>Dataset 4 (13)
3.	Human rhinovirus (HRV)	47	HRV	Dataset 1 (22)<br>Dataset 2 (25)
4.	Respiratory syncytial virus (RSV)	10	RSV	Dataset 1
5.	Influenza A virus	41	Influenza A	Dataset 2
6.	Influenza A virus and human coronavirus 229E	1	Other viruses	Dataset 2
7.	Influenza A virus and human rhinovirus	13	Other viruses	Dataset 2
8.	Influenza A virus and human coronavirus OC43	1	Other viruses	Dataset 2
9.	Influenza A virus and respiratory syncytial virus B	1	Other viruses	Dataset 2
10.	Influenza B virus	4	Other viruses	Dataset 2
11.	Influenza B virus and human rhinovirus	5	Other viruses	Dataset 2
12.	Human coronavirus HKU1	1	Other viruses	Dataset 2
13.	Human rhinovirus and enterovirus	1	Other viruses	Dataset 2
14.	Human rhinovirus and human coronavirus HKU1	2	Other viruses	Dataset 2
15.	Human rhinovirus and human coronavirus NL63	1	Other viruses	Dataset 2
16.	Human rhinovirus and respiratory syncytial virus A	1	Other viruses	Dataset 2
17.	Enterovirus	1	Other viruses	Dataset 2
18.	Human coronavirus NL63	1	Other viruses	Dataset 2
19.	Respiratory syncytial virus A	1	Other viruses	Dataset 2
20.	Subjects showed RVI infection, but no virus or virus subtype detected	22	Infected but no virus subtype detected	Dataset 2
